# Physiological Adaptations to Progressive Endurance Exercise Training in Adult and Aged Rats: Insights from the Molecular Transducers of Physical Activity Consortium (MoTrPAC)

**DOI:** 10.1093/function/zqae014

**Published:** 2024-03-28

**Authors:** Simon Schenk, Tyler J Sagendorf, Gina M Many, Ana K Lira, Luis G O de Sousa, Dam Bae, Michael Cicha, Kyle S Kramer, Michael Muehlbauer, Andrea L Hevener, R Scott Rector, John P Thyfault, John P Williams, Laurie J Goodyear, Karyn A Esser, Christopher B Newgard, Sue C Bodine, The MoTrPAC Study Group, Joshua N Adkins, Brent G Albertson, David Amar, Mary Anne S Amper, Euan Ashley, Dam Bae, Marcas M Bamman, Jerry Barnes, Bryan C Bergman, Daniel H Bessesen, Sue C Bodine, Thomas W Buford, Charles F Burant, Michael Cicha, Gary R Cutter, Luis Gustavo Oliveria De Sousa, Karyn A Esser, Facundo M Fernández, David A Gaul, Yongchao Ge, Bret H Goodpaster, Laurie J Goodyear, Kristy Guevara, Andrea L Hevener, Michael F Hirshman, Kim M Huffman, Bailey E Jackson, Catherine M Jankowski, David Jimenez-Morales, Wendy M Kohrt, Kyle S Kramer, William E Kraus, Sarah J Lessard, Bridget Lester, Malene E Lindholm, Ana K Lira, Gina Many, Nada Marjanovic, Andrea G Marshall, Edward L Melanson, Michael E Miller, Kerrie L Moreau, Venugopalan D Nair, Christopher B Newgard, Eric A Ortlund, Wei-Jun Qian, Blake B Rasmussen, R Scott Rector, Collyn Z-T Richards, Scott Rushing, Tyler J Sagendorf, James A Sanford, Irene E Schauer, Simon Schenk, Robert S Schwartz, Stuart C Sealfon, Nitish Seenarine, Lauren M Sparks, Cynthia L Stowe, Jennifer W Talton, Christopher Teng, Nathan D Tesfa, Anna Thalacker-Mercer, John P Thyfault, Scott Trappe, Todd A Trappe, Mital Vasoya, Matthew T Wheeler, Michael P Walkup, John P Williams, Zhen Yan, Jimmy Zhen

**Affiliations:** Department of Orthopaedic Surgery, School of Medicine, University of California San Diego, La Jolla, CA 92093, USA; Biological Sciences Division, Pacific Northwest National Laboratory, Richland, WA 99352, USA; Biological Sciences Division, Pacific Northwest National Laboratory, Richland, WA 99352, USA; Department of Internal Medicine, Carver College of Medicine, University of Iowa, Iowa City, IA 52242, USA; Department of Internal Medicine, Carver College of Medicine, University of Iowa, Iowa City, IA 52242, USA; Aging and Metabolism Research Program, Oklahoma Medical Research Foundation, Oklahoma City, OK 73104, USA; Department of Internal Medicine, Carver College of Medicine, University of Iowa, Iowa City, IA 52242, USA; Department of Internal Medicine, Carver College of Medicine, University of Iowa, Iowa City, IA 52242, USA; Department of Internal Medicine, Carver College of Medicine, University of Iowa, Iowa City, IA 52242, USA; Duke Molecular Physiology Institute, Duke University Medical Center, Durham, NC 27701, USA; Division of Endocrinology, Diabetes, and Hypertension, Department of Medicine, University of California, Los Angeles, CA 90095, USA; Research Service, Harry S. Truman Memorial Veterans’ Medical Center, Columbia, MO 65201, USA; NextGen Precision Health, University of Missouri, Columbia, MO 65201, USA; Department of Nutrition and Exercise Physiology, University of Missouri, Columbia, MO 65211, USA; Division of Gastroenterology and Hepatology, Department of Medicine, University of Missouri, Columbia, MO 65211, USA; Department of Cell Biology and Physiology, University of Kansas Medical Center, Kansas City, KS 66160, USA; KU Diabetes Institute, University of Kansas Medical Center, Kansas City, KS 66160, USA; Division of Aging Biology, National Institute on Aging, National Institutes of Health, Bethesda, MD 20898, USA; Section on Integrative Physiology and Metabolism, Joslin Diabetes Center, Harvard Medical School, Boston, MA 02215, USA; Department of Physiology and Aging, College of Medicine, University of Florida, Gainesville, FL 32610, USA; Duke Molecular Physiology Institute, Duke University Medical Center, Durham, NC 27701, USA; Department of Internal Medicine, Carver College of Medicine, University of Iowa, Iowa City, IA 52242, USA; Aging and Metabolism Research Program, Oklahoma Medical Research Foundation, Oklahoma City, OK 73104, USA

**Keywords:** training, treadmill, maximal oxygen uptake, body composition, citrate synthase, skeletal muscle, biorepository, aging

## Abstract

While regular physical activity is a cornerstone of health, wellness, and vitality, the impact of endurance exercise training on molecular signaling within and across tissues remains to be delineated. The Molecular Transducers of Physical Activity Consortium (MoTrPAC) was established to characterize molecular networks underlying the adaptive response to exercise. Here, we describe the endurance exercise training studies undertaken by the Preclinical Animal Sites Studies component of MoTrPAC, in which we sought to develop and implement a standardized endurance exercise protocol in a large cohort of rats. To this end, Adult (6-mo) and Aged (18-mo) female (*n* = 151) and male (*n* = 143) Fischer 344 rats were subjected to progressive treadmill training (5 d/wk, ∼70%–75% VO_2_max) for 1, 2, 4, or 8 wk; sedentary rats were studied as the control group. A total of 18 solid tissues, as well as blood, plasma, and feces, were collected to establish a publicly accessible biorepository and for extensive omics-based analyses by MoTrPAC. Treadmill training was highly effective, with robust improvements in skeletal muscle citrate synthase activity in as little as 1–2 wk and improvements in maximum run speed and maximal oxygen uptake by 4–8 wk. For body mass and composition, notable age- and sex-dependent responses were observed. This work in mature, treadmill-trained rats represents the most comprehensive and publicly accessible tissue biorepository, to date, and provides an unprecedented resource for studying temporal-, sex-, and age-specific responses to endurance exercise training in a preclinical rat model.

## Graphical Abstract

**Graphical Abstract:**
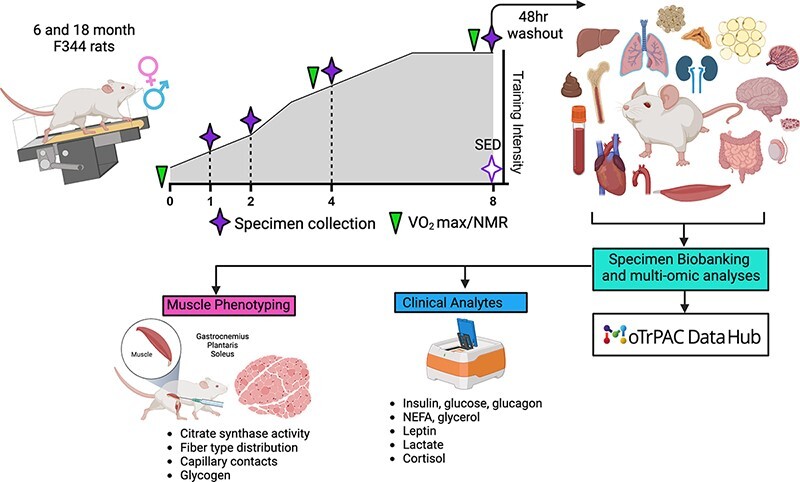

## Abbreviations

CoDA: compositional data analysis
CS: citrate synthase
CSA: cross-sectional area
F344: Fischer 344
GLM: generalized linear model
GLS: generalized least squares
ilr: isometric log-ratio
LG: lateral gastrocnemius
LMM: linear mixed-effects model
MG: medial gastrocnemius
MoTrPAC: Molecular Transducers of Physical Activity Consortium
MRS: maximum run speed
NEFA: nonesterified fatty acids
NIA: National Institute on Aging
NMR: nuclear magnetic resonance
OCT: optimal cutting temperature
PASS: Preclinical Animal Studies Sites
PL: plantaris
RT: room temperature
SED: sedentary (controls)
SOL: soleus
TD-NMR: time-domain NMR
VO_2_max: maximal oxygen consumption
WLS: weighted least squares

## Introduction

Endurance exercise training and habitual physical activity are cornerstones for improving or maintaining health and quality of life.^1–4^ Among its many benefits, regular exercise helps maintain independence later in life,^5^ reduces morbidity risk for over 26 chronic lifestyle-related diseases,^6–8^ and decreases all-cause mortality.^9^,^10^ While the beneficial effects of exercise are believed to extend across organ systems, only a few tissues, usually skeletal muscle and heart, have been studied in detail.^3^,^11–15^ Thus, remarkably, the collective impact of exercise training on molecular signaling across a broad range of tissues and, by extension, how regular exercise promotes health and reduces disease risk, is not well-defined.^16^,^17^ To address these gaps, through support from the National Institutes of Health Common Fund, the Molecular Transducers of Physical Activity Consortium (MoTrPAC) was established to develop an integrated molecular map of the adaptive response to exercise training across the lifespan. The primary goal is to provide a publicly available tissue biobank and multiomics data resource to support hypothesis-driven research.^18^

To better define the impact of exercise throughout the body, the Preclinical Animal Studies Sites (PASS) were established as one of the two exercise testing arms of MoTrPAC^18^ to complement its clinical study sites. Specifically, the objectives of the PASS were to (1) develop a standardized exercise protocol for the characterization of physiological adaptation to exercise and (2) collect an expansive group of tissues/organs for the creation of a publicly accessible tissue biorepository and multiomic analysis database. To meet these objectives, the Fischer 344 (F344) rat was chosen as the model organism. The rat has long been utilized to study the impact of endurance exercise training on biology and health.^19^ By way of its size, the rat also provides the capability to study a broad range of tissues, which have sufficient mass to allow molecular phenotyping on multiple platforms thus maximizing quality control and integration capabilities. Finally, given the genetic, physiological, and metabolic similarities between rats and humans,^20^ rats are a useful model of human phenotypic responses. To this point, rats have skeletal muscle fiber type distributions and glycogen utilization patterns more similar to humans than mice.^21^,^22^ The F344 rat strain is translationally relevant as it displays a proclivity toward insulin resistance and ectopic lipid deposition that increases with age,^23–25^ mimicking common aging phenotypes in humans impacted by endurance training.

Here, we describe the study design, physiological adaptations, and tissue acquisition after 1, 2, 4, or 8 wk of endurance exercise treadmill training at ∼70%–75% VO_2_max in a large cohort (*n* = 294) of male and female F344 rats that were 6 or 18 mo of age at the initiation of the study. Results from this study are designed to be used as a readily accessible database and biorepository resource for the scientific community to couple with current^26–29^ and future molecular profiling, thereby facilitating development of an integrative map of systemic adaptations to endurance training. Demonstrating the utility of this resource to the research community, we have recently undertaken multiomic analyses on 18 different tissues and the blood from a subset of the 6 month-old cohort of rats,^28^ including generating detailed insight into the molecular response in white adipose tissue,^26^ and the mitochondrial^29^ and nuclear transcription factor^27^ response across tissues.

## Methods

### Animals

**Figure 1.**
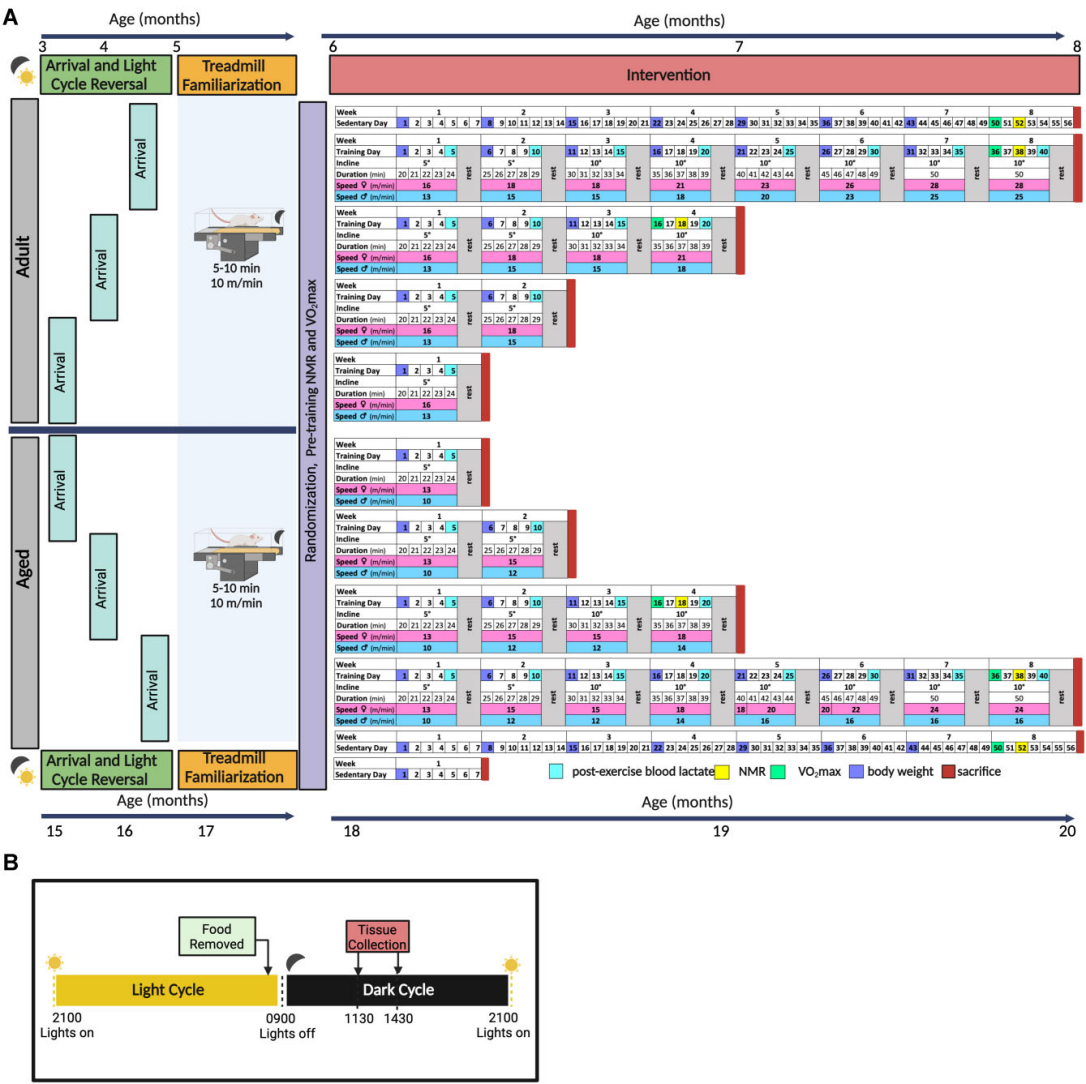
MoTrPAC PASS1B Study Overview and Design. (**A**) Overview of cohort intake, testing, and progressive endurance training protocol in male and female Adult and Aged F344 rats. Schematic displays pretraining acclimation and familiarization protocol for all rat cohorts. Note, postexercise blood lactate concentration was also measured on the first of each training week. Also, in the 18 mo cohort, an additional SED control group (for both sexes) was age-matched to 1 W training group; this group was included to account for potential aging effects. (**B**) Overview of the timeline of events on the day of sacrifice. This figure was created with BioRender.com (www.biorender.com) and confirmation of publication and licensing rights was obtained.

Male and female Fischer 344 (F344) inbred rats were obtained from the National Institute on Aging (NIA) rodent colony in cohorts of 20–30 rats. A total of 160 adult (3–5 mo of age) and 160 middle-aged (15–17 mo of age) rats were received at the animal test site (University of Iowa). There were five experimental groups for each age: sedentary control (SED) or 1, 2, 4, or 8 wk of treadmill training (1, 2, 4, and 8 W). To account for a potential effect of aging on outcome variables, an additional SED group was matched to the 1 W 18 mo group. The overall experimental design is outlined in Figure 1(A). The experiment was designed so that rats began exercise training at either 6 or 18 mo of age and were all housed at the test site for similar amounts of time (∼12 wk). We examined the response to training in these age groups because they represent adult rats with mature sexual and musculoskeletal organ systems and late middle-aged rats that have a low incidence of cancer and sarcopenia. Consequently, to meet the experimental design requirements, rats were delivered to the test site at different ages (Figure 1A; Table S1). Upon arrival, rats were placed into a reverse dark–light cycle housing with lights off at 9:00 am and lights on at 9:00 pm for a minimum of 10 d prior to familiarization to the treadmill. This allowed for training of the rats during their normal active period (dark phase), while also allowing for training to occur during normal working hours. This period of time was designed to provide sufficient time for the intrinsic circadian clocks across all tissues to entrain with the new light cycle. During this time, the rats were handled daily by the research staff to minimize stress. Rats of the same sex were housed two per cage (146.4 in^2^ of floor space) in ventilated racks (Thoren Maxi-Miser IVC Caging System) with Tekland 7093 Shredded Aspen bedding. Rats were fed a standardized pellet diet (Lab Diet 5L79) consisting of 64% carbohydrates, 21% protein, and 15% fat and given *ad libitum* access to food and water. Both the bedding and diet used are standard for this NIA rodent colony. Daily cage activity and food consumption were not measured. The animal housing room was monitored daily and maintained at 68°F–77°F and 25%–55% humidity. Red lights were used during the dark cycle to provide adequate lighting for staff to perform routine housing tasks, rodent handling, and exercise training; no standard lighting was used during the dark phase. All animal procedures were approved by the Institutional Animal Care and Use Committee at the University of Iowa.

### Treadmill Familiarization and Training

Treadmill exercise was performed on a Panlab 5-lane rat treadmill (Harvard Instruments, Model LE8710RTS). All animal handling and exercise was performed during the active phase (dark cycle) for nocturnal rodents. Following the initial acclimation period, rats went through a 12-d treadmill familiarization protocol (outlined in Table 1) to expose them to the treadmill and to identify noncompliant rats. Those rats that successfully completed the 12-d familiarization protocol and were judged to be compliant (score of 2–4) were entered into the MoTrPAC database and randomized into an experimental group. Noncompliant rats (score of 1) were removed from the study. The number of rats received, randomized into an experimental group, and completed the exercise training are provided in Table S1.

**Table 1.** Treadmill Familiarization Protocol

Days	Protocol
1–2	Rat was placed on the treadmill at a speed of 0 m/min for 10 min to familiarize it to the treadmill. The shock grid was blocked to prevent the rat from sitting on the grid.
3–5	Rat was placed on the treadmill with the shock grid blocked and ran at a speed of 6 m/min for 10 min. A pen with a dull point was used to gently prod the rat or turn its head to make it walk forward.
6–12	Rat was placed on the treadmill with the shock grid blocked and run at 10 m/min for 10 min. If the rat was unable to run at 10 m/min, the speed was reduced to 6 m/min and then increased to 8 mm/min for 5 min once the rat was able to run forward properly. On the next day the treadmill speed was set to 10 m/min. If the rat was unable to run at 10 m/min, the speed was reduced to 8 m/min. For those rats that were not able to run continuously at 10 m/min, a light shock was used to entice them to run. Rats were not allowed to sit on the shock grid. For those rats that liked to walk backwards, a pen was used to turn their head and prod them to run forward.
11	After the 10 min familiarization session, the rats were run for 2 min at a treadmill grade of 10° and a speed of 12 m/min.
12	Each rat was run on the treadmill at 0° incline and 10 m/min for 5 min after which the grade was increased to 10° and speed to 12 m/min for 5 min. Upon completion of the run, each rat was assigned a score ranging from 1 to 4, with 4 being the highest score. **Scoring criteria:** 4: rat is active on the treadmill the entire activity session without assistance. 3: rat required minimal assistance, defined as assistance for less than 25% of the time of the activity session. 2: rat required much assistance, defined as assistance for greater than 25% of the time of the activity session. 1: rat was noncompliant and failed to complete an activity session. Those rats that were unable to run on the treadmill for 5 min at a speed of 10 m/min and grade of 0° were classified as noncompliant and removed from the study.

Exercise training began at 6 or 18 mo of age and lasted for a duration of 1, 2, 4, or 8 wk. Rats were exercised on a motorized treadmill 5 d/wk using a progressive training protocol designed to elicit an intensity of ∼70%–75% of VO_2_max,^30^ The starting treadmill speed was based on VO_2_max measurements obtained following familiarization and 7–8 d prior to training in the compliant rats. Training was performed under red lights during the dark cycle and started no earlier than 10:00 am and no later than 5:00 pm over 5 consecutive days per week followed by 2 d of rest. Training was initiated with the treadmill set at a grade of 5° and a duration of 20 min. As illustrated in Figure 1(A), the duration of exercise was increased by 1 min each day until day 31 of training (start of week 7) when a final duration of 50 min was reached. The treadmill grade was increased from 5° to 10° at the start of week 3 and stayed at 10° for the remainder of the training. The starting treadmill speed varied as a function of sex and age and increased at the start of weeks 2 and 4–7. At the start of week 7, speed, grade, and duration were fixed and maintained for the final 10 d of the training intervention. If a rat was unable to perform at least 4 d of training per week, it was removed from the study and euthanized. Rats assigned to the Sedentary (SED) control group were placed on the treadmill for 15 min/d at 0 m/min for 5 d/wk and followed a schedule like the 8-wk training group. For insight into changes in body mass over time, at the beginning of each training week (including the first training session), body mass was measured in each rat immediately prior to beginning the treadmill session; rats were not fasted. Also, immediately after completing the first and fifth training session of each week, blood lactate concentration was measured via the tail vein (Lactate Plus meter). For each cohort, rats were exercised in groups of 5–6 animals per day, with the start day being staggered over 3–5 d.

### Body Composition

The minispec LF90II Body Composition Rat and Mice Analyzer (Bruker; 6.2 MHz Time-Domain Nuclear Magnetic Resonance [TD-NMR] system) was used for in vivo measurement of body fat, lean tissue (ie, fat-free mass, which includes skeletal muscle), and fluid in conscious animals. Pretraining body composition was determined 13 d prior to the start of training in all rats. Post-training body composition was determined for rats in the 4- and 8-wk training groups, and in the and 8-wk control (SED) group 5 d prior to tissue harvesting.

### Maximum Oxygen Consumption (VO_2_max)

VO_2_max testing was performed 7–8 d prior to the onset of training in all rats and during the last week of training for the 4- and 8-wk exercise groups. Maximum running speed (MRS), which was defined as the highest recorded speed, was recorded in all rats during the VO_2_max test. After completing the treadmill familiarization period, rats were acclimated to a single-lane enclosed treadmill (Columbus Instruments Metabolic Modular Treadmill) 2 d prior to testing. On the first day of acclimation, each rat was placed in the enclosed treadmill for 10 min at 0 m/min to acclimate them to the environment. On the second day of acclimation, the rat was placed in the enclosed treadmill for 10 min and ran at a speed of 10 m/min. On the test day, the rat was placed in the treadmill, and testing began once oxygen consumption had stabilized. Testing began with a warm-up for 15 min with the treadmill set at a speed of 9 m/min and 0° incline. Following the warm-up period, the incline was increased to 10° and treadmill speed was increased by 1.8 m/min every 2 min^30^; the protocol used differed by sex and training duration, with each protocol overviewed in Figure S1(A)–(F). During the test, shock was used sparingly, and only when the rat stopped running and sat on the shock area. Testing stopped when the rat sat on the shock area three consecutive times and did not respond to increased shock. Upon cessation of the test, the rat was removed from the enclosure and blood drawn from the tail vein to measure lactate. Criteria for reaching VO_2_max during this graded treadmill test was a plateau in oxygen uptake despite increased workload, a respiratory exchange ratio ≥ 1.05, and/or a nonhemolyzed blood lactate concentration ≥ 6 mmol/L (Lactate Plus).^30^ In Adult rats, VO_2_max and MRS was calculated at baseline for SED and 1, 2, 4, and 8 W training groups and post-training only in the SED and 4- and 8-wk training groups. In Aged rats, VO_2_max and MRS testing was performed at baseline and after training in the SED, 4 and 8 W training groups. Due to an equipment issue, the VO_2_max test was not performed in the 1 and 2 W groups, and only the MRS was recorded in the 4 W group pre- and post-training. A lactate of ≥ 6 mmol/L was recorded in 98% of the Adult rats and 85% of the Aged rats. An RER ≥ 1.0 was measured in 96% of the Adult rats and 83% of the Aged rats.

### Tissue Collection

Tissues were collected from all rats 48 h following the last training session. The duration between the last training session and tissue collection was chosen to focus on the cumulative effects of steady state treadmill training and to limit potentially confounding effects of the last acute exercise bout. On the day of collection, food was removed at 8:30 am, 3 h prior to the start of dissections, which occurred between 11:30 am and 2:30 pm (in the dark cycle) (Figure 1B). For each experimental cohort, dissections occurred during this 3 h window in 5–6 animals per day and over a period of 3–5 d. This design was chosen to limit potential effects of time-of-day and circadian oscillations.

**Figure 2.**
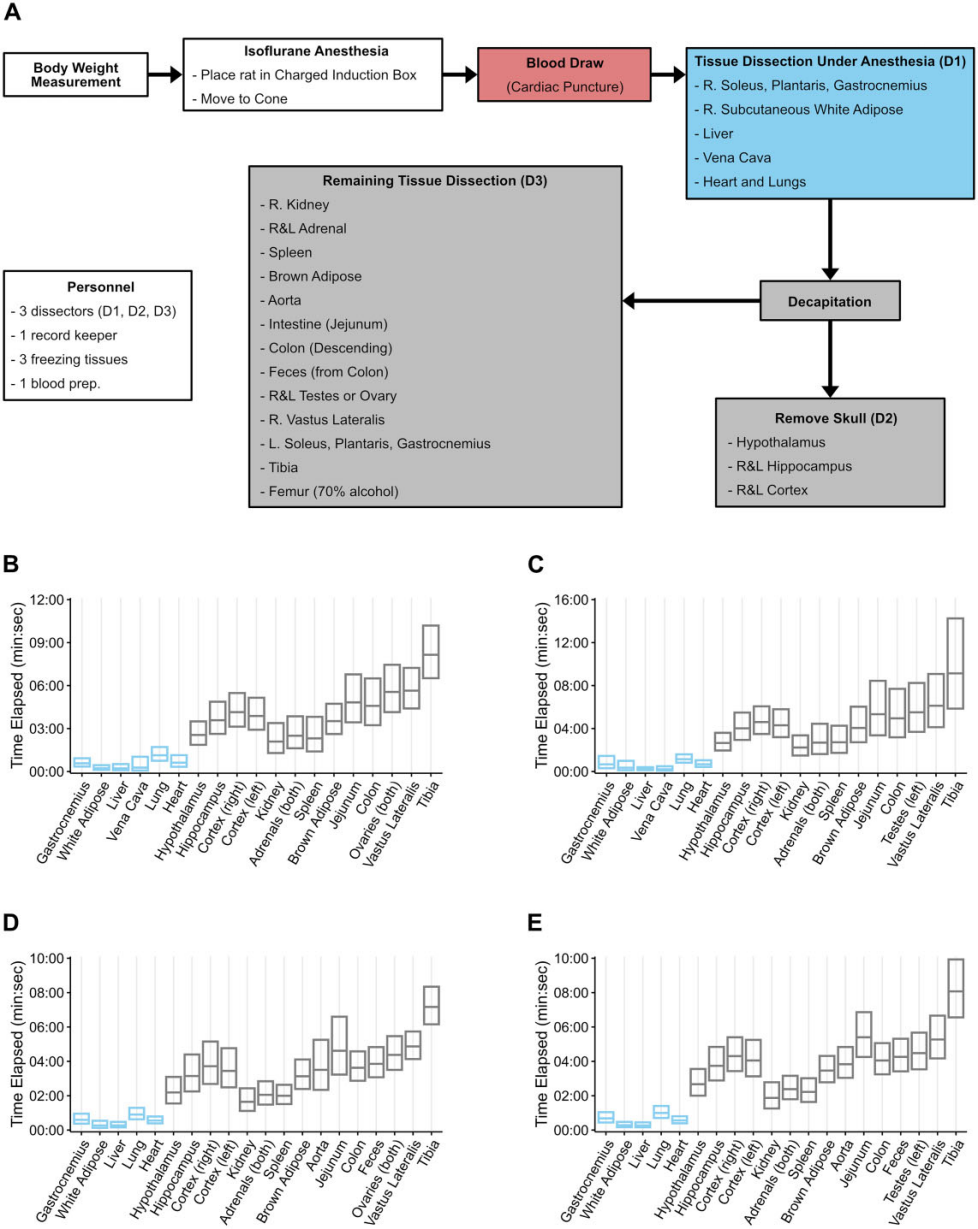
Dissection. (**A**) Tissue dissection workflow. Time elapsed from tissue collection (gastrocnemius, white adipose, liver, vena cava, lung, heart) or death to freezing for, (**B**) Adult females, (**C**) Adult males, (**D**) Aged females, and (**E**) Aged males. Boxes are mean ± 2 SD (calculated from the log-transformed times and back-transformed).

An overview of the workflow for tissue dissection is provided in Figure 2(A). Specifically, body weight was measured and then rats were placed in an induction box and sedated with inhaled isoflurane (3%–4%); rats were maintained in the dark until they were anesthetized, after which they were exposed to standard lighting. Once sedated, the rat was moved to a nose cone and continuously sedated with isoflurane (1%–2%). Blood was drawn via cardiac puncture followed by dissection of the right soleus (SOL), gastrocnemius, and plantaris (PL) muscles, right lateral subcutaneous (inguinal) white adipose tissue, right lobe of the liver, vena cava, and finally the heart and lungs. Removal of the heart was recorded as time of death. Immediately following removal of the heart, a guillotine was used for decapitation. The brain was removed from the skull and the hypothalamus, right and left hippocampus, and right and left cerebral cortex were collected, in order. Following decapitation, specific organs were removed from the body in the following order: right kidney, right and left adrenal, spleen, brown adipose (between shoulder blades), small intestine (jejunum), colon (transverse and descending) and feces, right testes or ovaries, right vastus lateralis, left SOL, gastrocnemius, and PL muscles, right tibia, and right femur. All tissues, except the left hindlimb muscles and femur, were flash frozen in liquid nitrogen immediately upon removal, placed in cryovials, and stored at −80°C. The left SOL, PL, medial gastrocnemius (MG), and lateral gastrocnemius (LG) muscles were removed, weighed, pinned to cork, frozen in chilled isopentane, and stored at −80°C for histological analysis. The femur was placed in 70% ethanol and stored at 4°C. The time of removal and freezing was recorded for all tissues. The average time between tissue removal and freezing or death (heart removal) and freezing are provided for each tissue in Figure 2(B)–(E). All tissues were subsequently shipped in dry ice to the biorepository at the University of Vermont for long-term storage and distribution.

### Muscle Fiber-Type Distributions and Fiber Size

Fiber-type percentages (based on myosin heavy chain expression) and fiber-type specific cross-sectional area (CSA) were determined in the SOL, PL, LG, and MG muscles for the SED and 8 wk training groups of both sexes and ages (48 animals per muscle). The SOL is a predominantly slow muscle in the rat with > 85% type I fibers, while the PL, MG, and LG are muscles that express all four fiber types with a predominance of fast (type II) fibers.^31^,^32^ Specifically, a portion of each frozen muscle was cut from the mid-belly, mounted on cork in embedding medium (OCT), and frozen. Care was taken during the blocking of the tissue to ensure that the muscle remained frozen. Serial sections (10 µm) were cut from the frozen tissue block using a rotary microtome in a cryostat (Leica CM1850 Cryostat, Germany) at −20°C and placed on glass slides. Sections were fixed for 5 min in cold acetone at −20°C. After the fixation step, sections were allowed to warm to room temperature (RT) for 5 min and then washed one time for 5 min in PBST (PBS + 0.1% Tween 20). Next, sections were incubated in blocking solution (BS) (5% Normal Horse Serum PBST) for 30 min at RT and then incubated at 4°C overnight with a cocktail containing antibodies against myosin heavy chains (MHC) and laminin: anti-MHC I (BA-F8), anti-MHC 2A (SC-71 s), anti-MHC 2B (BF-F3s) (Developmental Studies Hybridoma Bank, Iowa City, IA, USA), and antilaminin (L9393 Sigma). Antibodies were diluted in BS at 1:250 (MHC) and 1:500 (laminin). Following the overnight incubation, samples were washed 3 times for 5 min with PBST and then incubated in BS for 30 min at RT with fluorescently conjugated secondary antibodies (Alexa Fluor^TM^ ThermoFisher): goat antimouse IgG2B (A21242) to MHCI (1:250 dilation, excitation 647), goat antimouse IgG1 (A21127) to MHCIIa (1:500 dilation, excitation 555), goat antimouse IgM (A21042) to MHCIIb (1:500 dilation, excitation 488), and antirabbit IgG (H + L) (A31556B) to laminin (1:250 dilation, excitation 405). Samples were then washed 3 times for 5 min in PBST and covered with ProLong^TM^ Diamond Antifade Mountant (P36930; Thermo Fisher Scientific). For each muscle, the entire cross-section was digitally scanned at 10X objective on a Zeiss LSM710 confocal microscope using the Tile Scan tool. Images were collected within 5 d of staining. Fiber size and fiber type (MHC composition) of all fibers in the section was measured using Myovision 2.0 analysis software (Myovision 2.0 software, University of Kentucky).^33^

### CD31/PECAM 1—Capillary Contacts

Capillary contacts, the number of capillaries surrounding a single fiber, were determined in the SOL, PL, LG, and MG muscles for the SED and 8 wk training groups of both sexes and ages (48 animals per muscle). Serial cross-sections were cut and processed as described above. For each muscle, all slides were processed on the same day. Following the initial blocking step, samples were incubated with CD31/PECAM1 (R&D System AF3628) and dystrophin (H5) primary antibodies conjugated with Alexa Fluor 488 (Santa Cruz sc-365954, 1:80), diluted in BS, and incubated at 4°C overnight. On the following day, samples were washed 3 times for 5 min with PBST and then incubated for 50 min in BS at RT with the secondary antibody (555 IgG donkey antigoat (A21432, 1:500). Samples were then washed 3 times for 5 min in PBST and covered with ProLong^TM^ Diamond Antifade Mountant (P36930; Thermo Fisher Scientific).

For each muscle, the entire cross-section was digitally scanned at 10X objective using a Zeiss LSM710 confocal microscope using the Tile Scan tool. Images were collected within 5 d of staining. Images were quantified using ImageJ analysis software version 1.53r (National Institutes of Health, USA).^34^ Muscle fibers were analyzed from 2 to 3 regions of the cross-section: 3 regions from the LG, and 2 regions from the MG, PL, and SOL (scaling). Regions (0.83 µm × 0.83 µm) were selected to capture a diverse set of fiber types within each muscle. The number of capillaries surrounding each fiber (capillary contacts) was counted manually by a single individual who was blinded to the age, sex, and group of the sample. The mean ± SD number of fibers sampled per muscle was 367 ± 72 for the SOL, 390 ± 67 for the PL, 815 ± 89 for the MG and 1110 ± 184 for the LG.

### Citrate Synthase

Citrate Synthase (CS) activity was assayed using a modified protocol from Srere et al.^35^ The assay buffer (200 µL final volume) contained monobasic and dibasic potassium phosphate buffers (36.5 m m and 63.5 m m, respectively), EDTA (10 m m), DTNB (0.1 m m), acetyl-CoA (0.1 m m), and Triton X-100 (0.1% v/v). The reaction was initiated by the addition of 4 µL of muscle lysate (8 µg) and 5 µL of oxaloacetate (10 m m). Absorbance at 412 nm (25°C) was measured at 5 min. Values were then normalized to protein content and compared to a standard curve made with purified CS (Sigma, C3260).

### Glycogen

Glycogen was assessed using Glycogen Assay kit (Sigma, MAK016). Briefly, muscles were homogenized in 100 µL of water, boiled for 5 min, and centrifuged at 13 000 *g* for 5 min to remove debris. A volumef 10 µL of the supernatant were used in the assay following the kit protocol. An endpoint absorbance was measured at 570 nm. Results were analyzed by doing a background correction and normalized to milligram of tissue.

### Plasma Clinical Analytes

Using all Adult samples and only those Aged samples that were selected for multiomic analysis,^26–29^ a set of nine common clinical analytes was measured in plasma: glucose, lactate, glycerol, total ketones, nonesterified fatty acids (NEFA), glucagon, insulin, leptin, and corticosterone. The first five were measured using a Beckman DxC 600 clinical analyzer with reagents from Beckman (Brea, CA) and Fujifilm Wako (Osaka, Japan; total ketones and NEFA), while the others were measured in immunoassays using commercial kits from Meso Scale Discovery (Rockville, MD) and Alpco (Salem, NH; corticosterone). Catalog numbers are provided in Table S2.

### Statistical Analyses

The R programming language^36^ (v4.3.1) was used to perform all statistical analyses and generate most figures. The *emmeans* ^37^ (v1.8.8), *nlme* ^38^ (v3.1.163), and *tidyverse* ^39^ (v2.0.0) R packages formed the core of what was used.

#### Body Composition and Maximal Oxygen Consumption

##### (Post–Pre) Training Differences

For measures that were recorded both pre- and post-training—body mass recorded on the same day as the NMR body composition measures, NMR lean mass, NMR fat mass, and absolute and relative VO_2_max—we fit ordinary or weighted least squares (WLS) regression models with age, sex, group, and their interactions as predictors of the (post–pre) differences. Reciprocal group variances (calculated from each combination of age, sex, and group) were used as weights in the WLS models to account for observed heteroscedasticity. A few influential observations were removed and noted in the results. Then, two-sided *t*-tests were performed to determine whether the mean of the (post–pre) differences from each group was different from 0. That is, if there was a change from pre- to post-training measures. Since maximum run speed was recorded in 1.8 m/min intervals and could only take on a few distinct values, we instead performed nonparametric Mann–Whitney *U* tests^40^ separately for each combination of age, sex, and group. For all measures, *P*-values were adjusted across groups within each age and sex combination using the Holm procedure^41^ to control the family-wise error rate. Results of these analyses are provided in Table S3.

##### Baseline (Pretraining) Differences:

For all measures except maximum run speed, we fit log-link Gaussian, quasi-Poisson (% fat mass), or gamma (absolute and relative VO_2_max) generalized linear models^42^ (GLMs) with age, sex, group, and their interactions as predictors of the baseline (pretraining) values. GLMs can address nonconstant variance observed in strictly positive data, like VO_2_max. If data was not recorded for some age and group combinations, as with 4 W VO_2_max in the Aged animals, we instead concatenated age and group to form a single factor (*age_group*) and fit a model with predictors *age_group*, sex, and their interaction to avoid inestimable regression coefficients. For each Gaussian GLM, reciprocal group variances (calculated from the untransformed response values within each combination of age, sex, and group) were included as weights to account for any residual heteroscedasticity. Model parsimony was achieved through ANOVA *F*-tests and examination of regression diagnostic plots. Then, each of the trained timepoints were compared against their age- and sex-matched SED controls using the Dunnett multiple comparison procedure.^43^ If *age_group* was included as a predictor, comparisons were manually specified, and *P*-values were instead adjusted within each age and sex combination using the Holm procedure.^41^ Since the log link was used for all models, results are presented as ratios of trained to SED group means (fold-change).

Since maximum run speed was recorded in 1.8 m/min intervals and could only take on a few distinct values, we instead performed nonparametric Mann–Whitney *U* tests^40^ to compare each trained group to their matching control group. *P*-values were adjusted across comparisons within each sex and age combination using the Holm procedure.^41^ Results of these analyses are provided in Table S4 (“NMR & VO_2_max” tab).

#### Weekly Body Mass

Weekly body mass was recorded prior to the beginning of each week from weeks 1 to 8. We filtered the data to only those observations collected from the SED and 8 W groups, since this allowed for the most weekly comparisons. Since there are longitudinal measures from each rat, we used the *nlme:: gls* ^38^  *R* function to fit a generalized least squares (GLS) model^44^ with age, sex, group, week (categorical: 1–8), and their interactions as predictors of log(body mass). Since the correlations between measurements from the same rat decrease as the time lag (number of weeks between the measurements) increases, the correlation structure was specified with *nlme: corAR1(form = ∼ 1 | pid)*, where *pid* is a unique identifier for each rat. Model parsimony was achieved via likelihood-ratio tests. Then, we tested whether the mean of the 8 W group was different from the mean of the SED group at each week. *P*-values were adjusted across weeks 1–8 within each combination of age, sex, and group (SED or 8 W) using the Holm procedure.^41^ Results of this analysis are provided in Table S4 (“Weekly Body Mass” tab).

#### Fiber-Type-Specific Measures

*Cross-sectional area*: Since the SOL only consists of two of the four fiber types (types I and IIa), we first created a new categorical variable called *muscle_type* by concatenating muscle and fiber type to avoid inestimable regression coefficients. Since there are repeated measures from each animal, we fit a linear mixed-effects model (LMM) with age, sex, group (SED or 8 W), *muscle_type*, and their interactions as predictors of the log-transformed fiber type-specific CSA with a random intercept for each rat. Precision weights were specified with *nlme:: varIdent(form = ∼ 1 | muscle_type)* to account for heteroscedasticity. Then, we tested whether the mean of the 8-wk-trained group was different from that of the SED control group for each combination of age, sex, group, and *muscle_type. P*-values were Holm-adjusted^41^ across all 2 (SOL only) or 4 fiber types within each age, sex, and muscle combination. Since the response was log-transformed, results are presented as ratios of 8 W to SED group means (fold-changes) in Table S4 (“Mean Fiber Area” tab).

*Fiber-type distribution:* We performed compositional data analysis (CoDA)^45–47^ of the fiber counts, which we believe is more appropriate than common statistical methods for assessing fiber-type distribution. CoDA is appropriate for positive data carrying relative, rather than absolute, information, and it is used extensively in the geosciences (eg, analysis of mineral compositions).^48^ Additionally, since each set of fiber-type proportions are derived from the same animal and must necessarily sum to 1, a change in one fiber-type proportion would affect the remaining proportions. This violates the independence of observations assumption of classic analysis approaches like ANOVA, necessitating a different approach—CoDA.

Data preparation began by converting the fiber counts from each rat to proportions using the total number of fibers per muscle. Then, we applied the isometric log-ratio (ilr) transformation,^49^ which uses the sequential binary partitions {I ||| IIa || IIx | IIb} to generate balances b1, b2, and b3.^47^^(pp107–108)^,^50^

1. b1 = $\frac{1}{{\sqrt 2 }}log\big( {\frac{I}{{g( {IIa,{\mathrm{\ }}IIx,{\mathrm{\ }}IIb} )}}} \big)$ or $log\big( {\frac{I}{{IIa}}} \big)$ (SOL)
2. b2 = $\sqrt {\frac{2}{3}} log\big( {\frac{{IIa}}{{g( {IIx,{\mathrm{\ }}IIb} )}}} \big)$
3. b3 = $log\big( {\frac{{IIx}}{{IIb}}} \big)$

where *g*(·) denotes the geometric mean (the *n*th root of the product of *n* values: a measure of centrality) of the subcomposition. These partitions were chosen for the following interpretations of their balances:

1. b1 = type I compared to {IIa, IIx, IIb} fibers
2. b2 = type IIa compared to {IIx, IIb} fibers
3. b3 = type IIx compared to type IIb fibers

Reducing the compositions in the simplex 𝒮^4^ to ilr coordinates in ℝ^3^ and the two-component composition (SOL) in 𝒮^2^ to values along the real number line ℝ avoids singularity of the variance–covariance matrix, which would present problems for the statistical analyses that we will describe, though we necessarily sacrifice some interpretability of the results. The matrix of column vectors (the balances) follow a multivariate Normal distribution, so, for each muscle, we fit a multivariate multiple regression model with categorical variables age, sex, group, and their interactions as predictors of the 1 (SOL) or 3 (LG, MG, and PL) dependent variables.

For all muscles, we utilized *t*-tests to compare the mean of each balance from the 8 W group to the corresponding mean from the SED group (eg, ${{\underline {b1} }_{8W}} - {{\underline {b1} }_{SED}}$). The resulting *P*-values were Holm-adjusted^41^ across the muscles within each combination of age and sex. The differences between balances are not easily interpretable, so results are presented as a shift between specific fiber types (*Muscle Fiber Type Distribution* Results). Results of these analyses are provided in Table S4 (“Fiber Count” tab).

#### Muscle Morphology and Biochemistry

For each muscle-specific measure—capillary contacts, CS activity, glycogen, mean CSA, and terminal mass—we examined their mean-variance relationship. Informed by these relationships, we fit LMMs with log-transformed or square-root-transformed (glycogen only) dependent variables. Variables age, sex, group, muscle, and their interactions were included as predictors with a random intercept for each rat. LMMs were utilized because there are repeated measures for each rat, which violates the independence assumption of ordinary linear regression, while an appropriate variance-stabilizing transformation was applied to each response to address heteroscedasticity. If heterogeneity of the residuals was still observed, weights were included with *nlme:: varIdent*. Finally, model parsimony was achieved through ANOVA *F*-tests and examination of regression diagnostic plots. Next, we compared each of the trained timepoints against their age- and sex-matched SED controls using the Dunnett multiple comparison procedure^43^ within each muscle. Since glycogen did not use a log-transform, we instead estimated the marginal means on the square-root scale, back-transformed to the original glycogen concentration scale while adjusting for bias, and then log-transformed these values before setting up the contrasts. In doing so, we are able to present results as ratios of trained to SED group means (fold-changes), as with the other muscle measures. Results of these analyses are provided in Table S4 (“Muscle Measures” tab).

#### Clinical Analytes

For each of the plasma clinical analytes, we first examined their mean–variance relationship and fit an appropriate log-link GLM assuming the data followed a Gaussian (corticosterone, glucose, insulin, lactate, and leptin), quasi-Poisson (glucagon), gamma (glycerol and total ketones), or inverse Gaussian (NEFA) distribution. GLMs can address the issue of nonconstant variance typically observed in strictly positive data. Reciprocal group variances (calculated from each combination of age, sex, and group) were included as weights in the Gaussian GLMs to account for remaining heteroscedasticity. Age, sex, group, and their interactions were included as predictors, and model parsimony was achieved through ANOVA *F*-tests and examination of regression diagnostic plots. Then, we compared each of the trained timepoints against their age- and sex-matched SED controls using the Dunnett multiple comparison procedure.^43^ Since the log link was used for all models, results are presented as ratios of trained to SED group means (fold-changes) in Table S4 (“Plasma Analytes” tab).

### Calculation of Percent Change

We do not perform regression analyses on the post/pre values for each rat, the results of which could easily be converted to % change with (post/pre–1) × 100, because the use of a ratio in regression analyses can lead to incorrect or misleading inferences.^51^ Instead, % change from pre- to post-training was calculated by dividing the differences in means (“(Post–Pre) Training Differences” Methods) by the corresponding pretraining means (“Baseline (Pretraining) Differences” Methods) and multiplying by 100%. For the trained vs. SED comparisons, % change from SED to trained was calculated by subtracting 1 from the ratios and multiplying by 100.

## Results

Baseline Phenotypes Across Cohorts: Given the scale of this study, rats arrived at the facility in different shipments. For insight into cohort matching, we first confirmed that the rats within each age, group, and sex were well-matched by comparing baseline (ie, pretraining) phenotypic parameters for VO_2_max and/or MRS, and body composition between the four training groups (1, 2, 4, and 8 W) and the SED controls.

*Baseline VO_2_max and MRS:* In Adult female rats, absolute VO_2_max was the same between all groups (Figure S2A). Relative VO_2_max was not different between the SED and trained groups, with the exception of an 8% lower relative VO_2_max in 1 W relative to SED Adult females (Figure S2B). Further, baseline MRS was similar across all groups (Figure S2C). In Adult male rats, absolute VO_2_max was 8% higher in 1 W compared to SED rats, with no significant differences in the other trained groups. The mean relative VO_2_max was 6% higher in 2 W relative to SED, but the other groups were not significantly different from SED (Figure S2D–E). Relative to SED, Adult male rat MRS was modestly higher in the 1 W and 2 W groups (Figure S2F). In Aged rats, VO_2_max was measured in the SED and 8-wk-trained groups, only (see “Methods”). In Aged female rats, the mean relative pretraining VO_2_max was 7% higher in the 8 W group relative to SED, though neither absolute VO_2_max nor MRS were different between the SED and 8 W groups at baseline (Figure S2G–I). In Aged male rats, no differences were observed in absolute or relative VO_2_max or MRS (Figure S2J–L). Together these findings suggest the cohorts of rats were well-matched.

*Body Mass and Composition*: Overall, body mass and body composition were well-matched across all cohorts upon arrival and prior to the beginning of training (Figure S3). In female and male Adult rats (Figure S3A–J), the greatest differences were found between the 1 and 2 W groups (which arrived as a single cohort) and the SED group, with total body mass in males and females being significantly higher in the 1 and 2 W groups relative to SED (6% and 5%, respectively) (Figure S3A). In Adult females, the greater body mass was due to increases in both whole-body fat and lean mass (Figure S3B–E). In Adult males, the greater body mass was largely due to an increase in fat mass (Figure S3F–J) at the beginning of the study. In Aged females and males (Figure S3K–T), body mass was modestly, but significantly, lower in female and male 1 W (−8% and 4%, respectively) and 2 W (both, −4%), which arrived as a single cohort, and 4 W (−6% and 4%, respectively), which arrived as a separate cohort, as compared to SED. The lower body mass in 1, 2, and 4 W Aged females was accompanied by a significantly lower lean mass (Figure S3M) and % lean mass (Figure S3O); there were no differences in fat mass (except for 1 W [−19%]; Figure S3L) or % fat mass (except for 4 W [−9%]; Figure S3N) in the trained groups, as compared to SED. Despite the changes in body mass, in Aged males, there were modest statistical differences in body composition between the five trained groups and SED; fat mass (Figure S3Q) and % fat mass (Figure S3S) were lower in 4 W (−7% and −5%, respectively), while % lean mass (Figure S3T) was higher in 1 W (+2%) and 2 W (+3%), as compared to SED.

Overall, these data show that while rats of both sexes arrived in separate cohorts, at different ages and across a period of 6 mo within each age, the major phenotypic physiological parameters were very well-matched across all groups prior to beginning the treadmill training intervention.

### Phenotypic Responses to Training

#### Progressive Endurance Exercise Training Protocol

To estimate running intensity during training, which was designed to target an exercise intensity of ∼70–75% VO_2_max, blood lactate concentration was measured from the tail vein at the completion of the first training bout each week. The week 4 values for the 4 W training group animals are the end-of-week postexercise blood lactate (day 20), since the VO_2_max testing was performed at the start of that week. In line with a targeted intensity of 70%–75% VO_2_max, the mean ± SD of the blood lactate concentration was 4.5 ± 1.6 m m and 3.4 ± 1.4 m m for Adult females and males and around 4.7 ± 2 m m and 3.5 ± 1.7 m m in Aged females and males, respectively (Figure S4A–D).

#### Effects of Training on VO_2_max and MRS

Treadmill testing was performed in pre- and post-training in the SED, 4 W, and 8 W groups of Adult and Aged rats to assess the adaptation to the progressive treadmill training.

**Figure 3.**
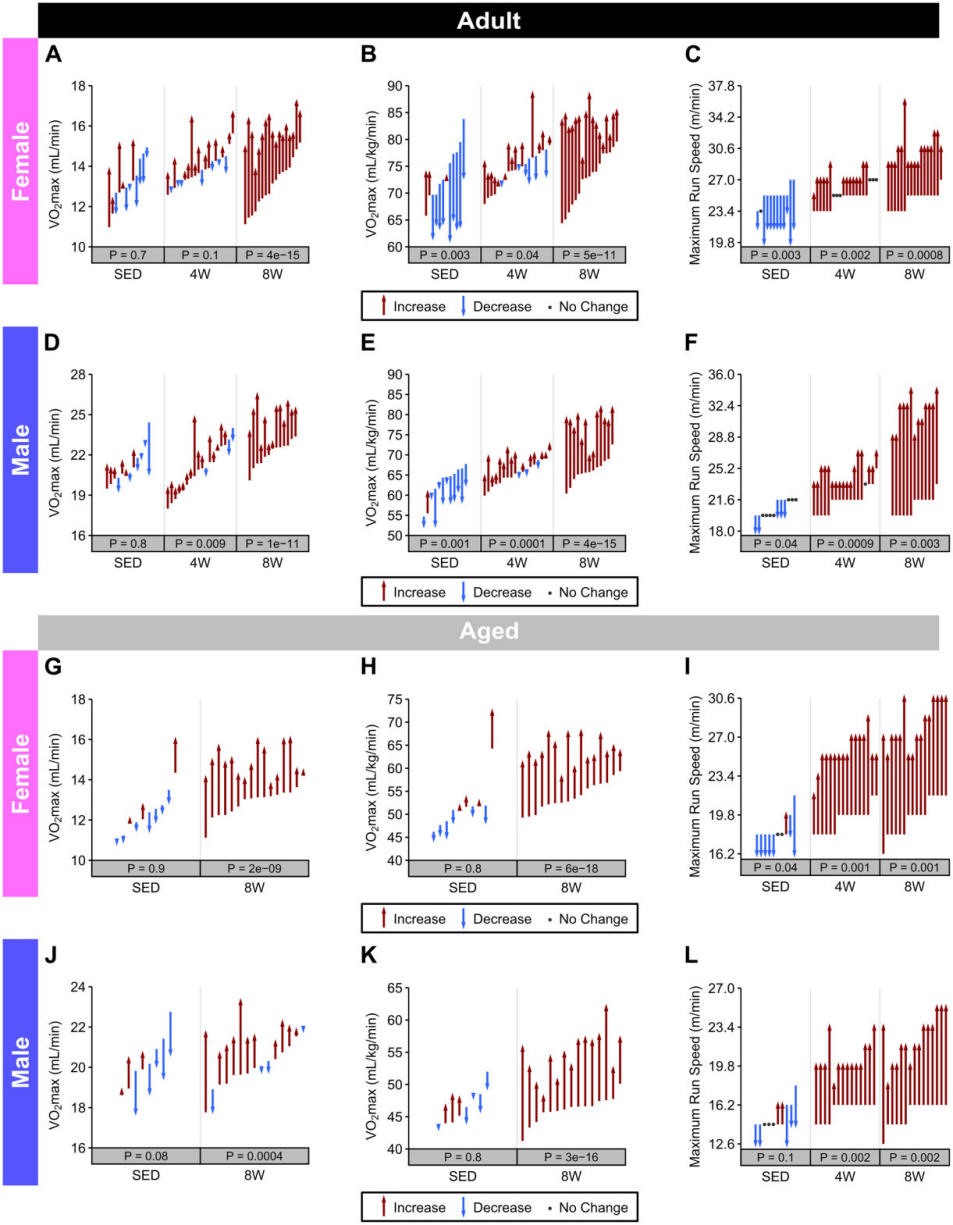
VO_2_max and MRS. Pre- and post-training measures of absolute VO_2_max, VO_2_max relative to total body mass, and MRS in Adult females (**A**)**–**(**C**), Adult males (**D**)**–**(**F**), Aged females (**G**)**–**(**I**), and Aged males (**J**)**–**(**L**). Each arrow or point represents a single rat, and they span from pre- to post-training values. Arrows are colored according to the direction of change from pre to post, and individual rats are arranged in ascending order by their pretraining value within each group. *P*-values were obtained from two-sided one sample *t*-tests of the (post–pre) differences, and they were Holm adjusted within each combination of age and sex.

*Adult group*. In the SED groups of female and male rats, absolute VO_2_max did not change over the 8-wk training period, though relative VO_2_max decreased by a mean of 6.9 (−9%) and 2.7 (−6%) mL/kg/min in females (Figure 3A and B) and males (Figure 3D and E), respectively. MRS also decreased significantly in the SED females and males (Figure 3C and F, respectively). In females in response to training, absolute VO_2_max was higher only in 8 W (+20%; (Figure 3A), while relative VO_2_max increased in both 4 W (1.8 mL/kg/min, +2%) and 8 W groups (10.2 mL/kg/min, +14%) (Figure 3B). MRS also increased in 4 and 8 W females (Figure 3C). Male rats displayed similar improvements in absolute (Figure 3D) and relative (Figure 3E) VO_2_max at 4 wk (absolute: +4%, relative: +4%) and 8 wk (absolute: +11%, relative: +17%), as well as a robust improvement in MRS (Figure 3F).

*Aged group*. In Aged rats, neither absolute nor relative VO_2_max changed significantly in the SED groups of either females (Figure 3G and H) or males (Figure 3J and K). Following 8 wk of training, both absolute and relative VO_2_max increased in Aged female (absolute: +15%, relative: +18%; Figure 3G and H, respectively) and male rats (absolute: +6%, relative: +18%; Figure 3J and K, respectively). MRS was measured at both 4 and 8 wk of training and increased significantly in both females (Figure 3I) and males (Figure 3L).

**Figure 4.**
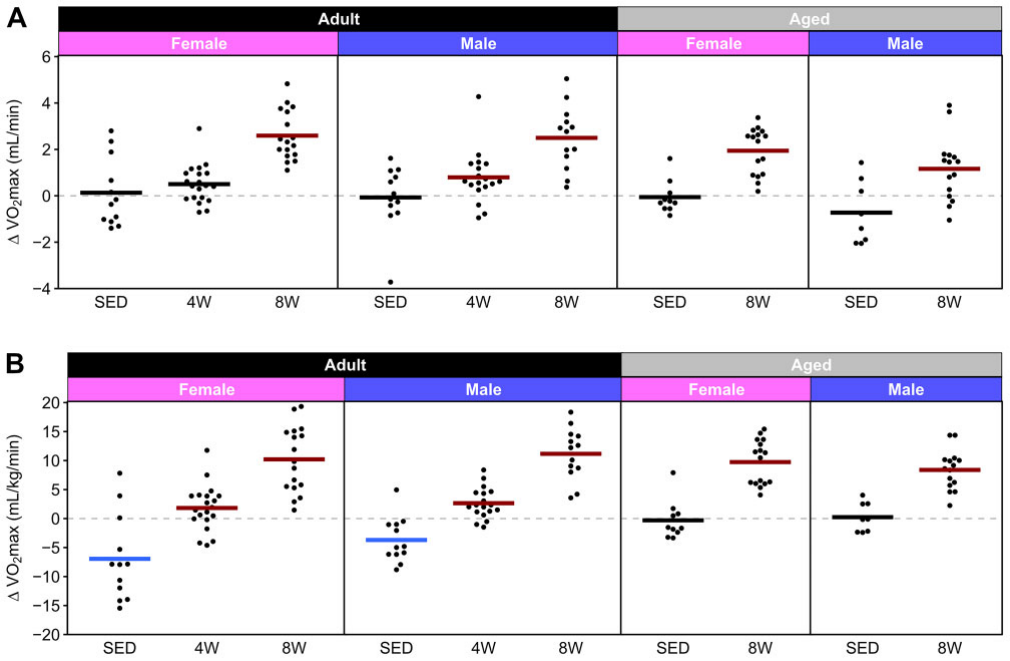
Delta VO_2_max. Change in absolute (**A**) and relative (**B**) VO_2_max from pre- to post-training. Horizontal lines represent the mean of each group. A colored line indicates that the mean of the group was significantly different from zero, while a black line indicates that the mean was not significantly different from zero. Exact *P*-values are shown in Figure 3.

The mean change in absolute (mL/min) and relative (mL/kg/min) VO_2_max as the result of training are plotted for all groups in Figure 4 and demonstrates that increases in both absolute and relative VO_2_max with training were similar across age and sex. Descriptive statistics (mean, SD, minimum, maximum, range, and coefficient of variation) for all groups for pre- and post-training are provided in Table S5 (absolute VO_2_max) and Table S6 (relative VO_2_max).

#### Changes in Body Mass and Composition With Training

In both Adult and Aged rats, pre- and post-training measures of body composition by NMR were taken in SED, 4 W, and 8 W rats. In addition, body mass was assessed at the beginning of each training week in all cohorts.

**Figure 5.**
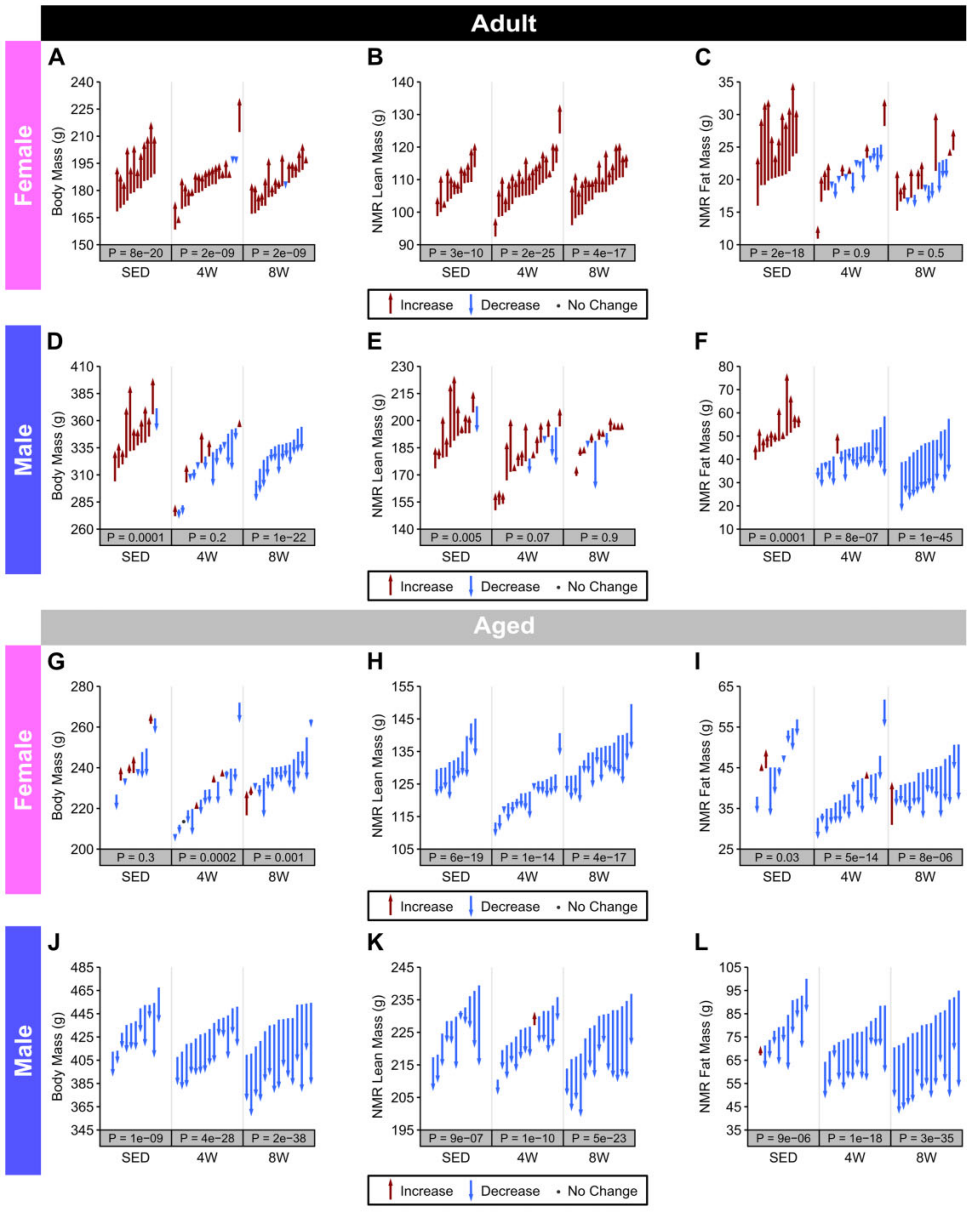
Body composition. Pre- and post-training measures of body composition (body mass, lean mass, and fat mass) in Adult females (**A**)**–**(**C**), Adult males (**D**)**–**(**F**), Aged females (**G**)**–**(**I**), and Aged males (**J**)**–**(**L**). Each arrow or point represents a single rat, and they span from pre- to post-training values. Arrows are colored according to the direction of change from pre to post, and individual rats are arranged in ascending order by their pretraining value within each group. *P*-values were obtained from two-sided one sample *t*-tests of the (post–pre) differences, and they were Holm adjusted within each combination of age and sex.

*Adult group*. In female rats, total body mass increased significantly in SED and the 4 W and 8 W training groups (Figure 5A). In SED females, such changes were accompanied by an increase in lean (+5%) and fat (+39%) mass (Figure 5B and C, respectively); accounting for changes in body mass, this translated to a decrease in % lean mass (−5%) and an increase in % fat mass (+11.5%; Figure S5A and S5B, respectively). With training, lean mass (Figure 5B) and % lean mass (Figure S5B) increased significantly in 4 W (+6% and +2%, respectively) and 8 W females (+7% and +2%, respectively). No training-induced changes in fat mass were observed in females (Figure 5C; Figure S5A). In SED males, body mass (Figure 5D), lean mass (Figure 5E), and fat mass (Figure 5F) were increased (+7%, +6%, and +17%, respectively). Accounting for changes in body mass, % fat mass increased by 9% (Figure S5D), though there were no changes in percentage lean mass (Figure S5E). In trained males, % lean mass increased by 5% at both 4 W and 8 W timepoints (Figure S5E), though there was no statistically significant change in absolute lean mass (Figure 5E). Overall, males decreased total fat mass (Figure 5D) and % fat mass (Figure S5D) at 4 W (total: −18%; %: −16%) and 8 W (total: −38%; %: −36%), whilst total body mass only decreased in 8 W (Figure 5D). Though we did not assess changes in body composition in the 1 W and 2 W groups, we did assess changes in body mass. In both 1 and 2 W females (Figure S5C) and males (Figure S5F), the terminal body mass was 1%–4% lower than pretraining body mass (which was measured on the NMR day).

*Aged group*. In Aged SED female rats, total body mass (Figure 5G) did not change, though lean mass (−6%; Figure 5H) and fat mass (−6%; Figure 5I) were both significantly decreased (Figure 5G–I). In trained Aged females, body mass decreased in 4 W (−2%) and 8 W (−3%) (Figure 5G). These changes paired with decreases in total lean mass (4W: −3%, 8W: −6%; Figure 5H), total fat mass (4W: −11%, 8W: −14%; Figure 5I), % fat mass (4W: −9%, 8W: −14%; Figure 5G), and % lean mass (4W: −1%, 8W: −3%; Figure S5H), in trained females. In males, total body mass decreased in 4 W (−6%), 8 W (−11%), and the SED group (−5%) (Figure S5J). The decrease in body mass in SED males was accompanied by a decrease in lean mass (−4%; Figure 5K) and fat mass (−12%; Figure 5L); these changes in total lean and fat mass resulted in a significant decrease in % fat mass (−8%; Figure S5J), while % lean mass was unchanged (Figure S5K). Decreases in body mass in the 4 and 8 W training groups were driven by decreases in total lean mass (4W: −3%; 8W: −7%; Figure 5K) and total fat mass (4W: −20%; 8W: −33%; Figure 5L), which resulted in a substantial decrease in % fat mass (4W: −15%; 8W: −25%; Figure S5J) and a modest, but significant, increase in % lean mass (4W: +3%; 8W: +5%; Figure S5K). In 1 and 2 W training groups, both males and females displayed a decrease in terminal body mass as compared to pretraining body mass (which was measured on the NMR day) (females: −3% and −5%, males: −5% and −6%) (Figure S5I).

**Figure 6.**
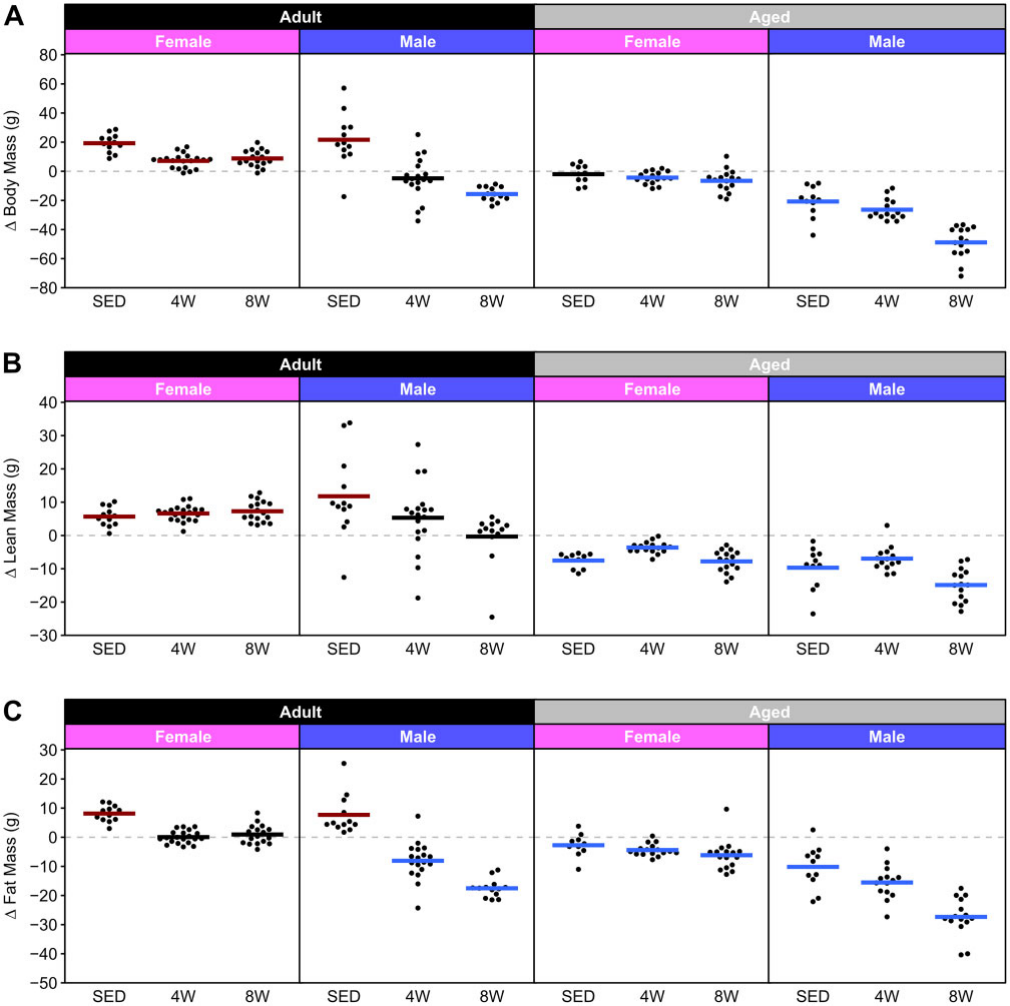
Delta body composition. Change in body mass (**A**), lean mass (**B**), and fat mass (**C**) from pre- to post-training. Horizontal lines represent the mean of each group. A colored line indicates that the mean of the group was significantly different from zero, while a black line indicates that the mean was not significantly different from zero. Exact *P*-values are shown in Figure 5.

The absolute changes in body mass, fat mass, and lean mass as the result of training are plotted for all groups in Figure 6. The data highlight differential responses of Adult males and females to exercise training, with males losing total body and fat mass; in contrast, trained females maintained a constant total body and fat mass and did not gain body mass like the SED group. In Aged rats, in contrast to Adults, trained males and females both display decreases in total body and fat mass. Descriptive statistics (mean, SD, minimum, maximum, range, and coefficient of variation) for all groups for pre- and post-training are provided in Table S7 (body mass), Table S8 (lean mass), and Table S9 (fat mass).

#### Weekly Monitoring of Body Mass

*Adult group*. In Adult females, body mass increased over time in the SED group and remained fairly constant in the 8 W group (Figure S6A). Conversely, the body mass of SED males remained constant and decreased over time in the 8 W group (Figure S6B). Compared to their age-matched SED counterparts at each week, the mean body mass of 8 W females was ∼4% lower starting at week 5 and remained ∼4% lower through week 8 (Figure S6E). In males, mean body mass of the 8 W animals was 5% lower than their age-matched SED counterparts starting at week 4; body mass continued to decrease with training duration until the difference in body mass was ∼9% at week 8; (Figure S6E).

*Aged group*. In Aged females, body mass remained constant over 8-wk in the SED group (Figure S6C). In the 8 W group, body mass decreased initially at the start of training before returning to week 1 values at the beginning of week 8 (Figure S6C). In SED males, there was an initial decrease in body mass followed by a plateau from weeks 3 to 8 (Figure S6D). In 8 W males, however, there was an immediate and consistent decrease in body mass that stabilized starting at week 7 (Figure S6D). Compared to their age-matched SED counterparts at each week, the mean body mass of 8 W males was lower at the beginning of week 4, with similar decreases in body mass in Aged and Adult males in response to training (Figure S6E). In Aged females, while not statistically significant, there was a 4% decrease at the start of week 4 that persisted through week 8, much like what was observed in the Adult females (Figure S6E).

### Endpoint Measures

#### Terminal Muscle Mass

Terminal masses for the four collected muscles are presented in Figure S7(A)–(P).

*Adult group*. In females, PL (Figure S7C) and SOL (Figure S7D) masses were significantly higher in the 1 W (SOL only: 8%), 2 and 4 W groups relative to the SED controls (PL: 4W: +8%, 8W: +6%; SOL: 2 W and 4W: +9%); this may relate to the fact that baseline body mass and lean mass of the 1 and 2 W cohorts were higher than the SED group (Figure S4A and S4C ; this could be because the 1 and 2 W rats were from a different cohort than the SED rats). In trained males, the LG (Figure S7E), PL (Figure S7G), and SOL (Figure S7H) were not different from SED at any timepoint. In the MG, muscle mass was lower (−7%) than SED at 4 W, only (Figure S7F).

*Aged group*. In Aged females, the MG (Figure S7J), PL (Figure S7K), and SOL (Figure S7L) mass in the 8 W group was significantly greater than SED, while there was no difference in the LG (Figure S7I); the mean % difference, as compared to SED, was 7%, 8%, and 11% for the MG, PL, and SOL, respectively. In Aged males, there were no significant differences in the terminal muscle masses of any trained groups, as compared to SED (Figure S7M–P).

### Muscle Fiber Types and FIber Type Specific CSA

**Figure 7.**
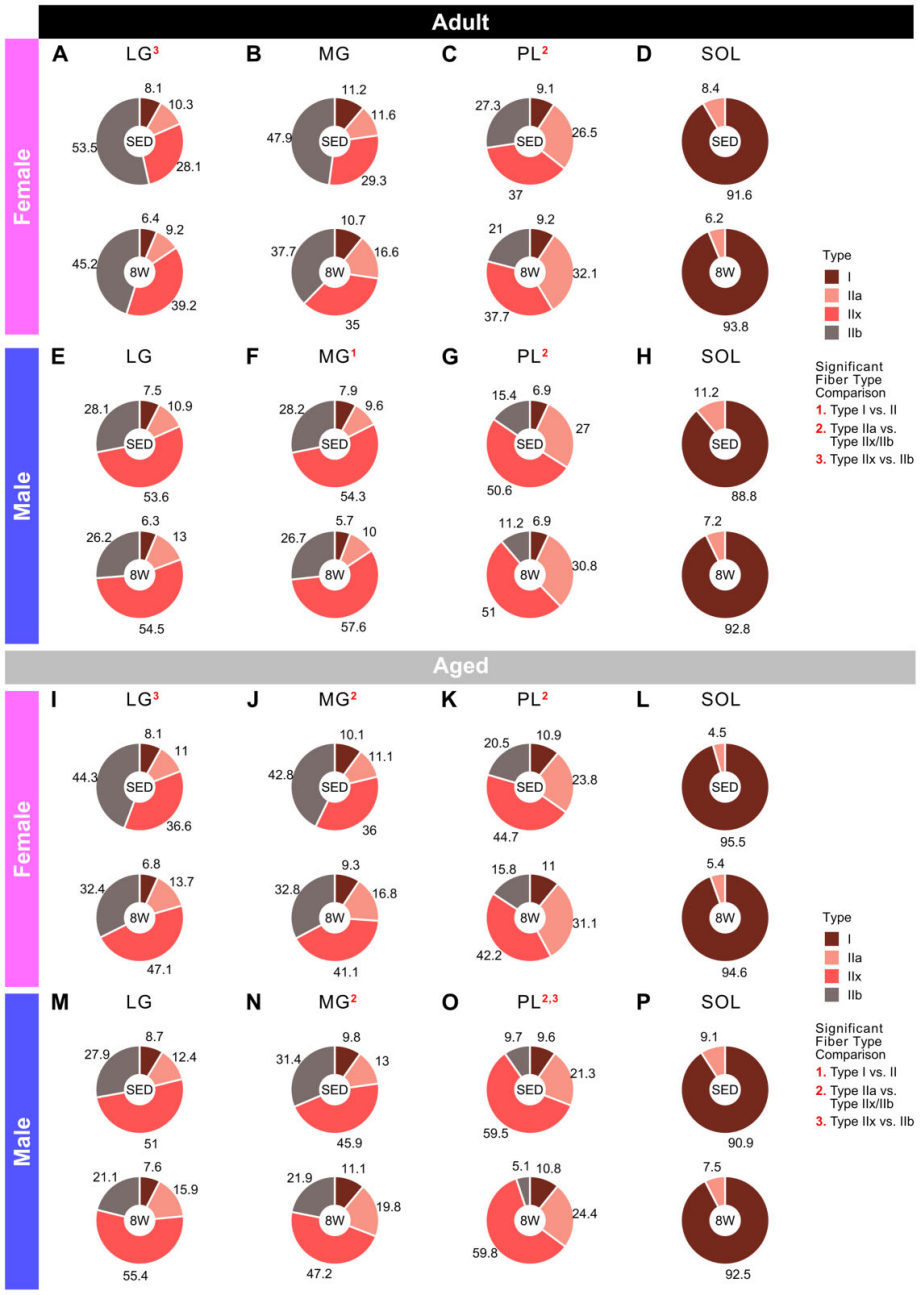
Mean fiber type %. Mean percentage of each fiber type (I, IIa, IIb, and IIx), determined by MHC expression, in the LG, MG, PL, and SOL of Adult females (**A**)**–**(**D**), Adult males (**E**)**–**(**H**), Aged females (**I**)**–**(**L**), and Aged males (**M**)**–**(**P**). Each donut chart summarizes measurements taken from 6 rats. Superscript numbers denote a significant difference (two-sided, two-sample *t*-test; Holm *P* < .05) between the 8 W and SED means for a particular fiber type ratio (described on the right of the figure and in the “Fiber-Type-Specific Measures: Fiber type distribution” Methods).

*Fiber type distributions*. In Adult females, there was a shift from type IIb to type IIx fibers in the LG with 8 wk of training (Figure 7A), and an increase in type IIa fibers relative to the type IIb and IIx fibers in the PL (Figure 7C), with no training-related differences in fiber type composition in the MG (Figure 7B) or SOL (Figure 7D). In Adult males, there were no training-related differences in fiber type composition in the LG (Figure 7E) or SOL (Figure 7H). In contrast, the ratio of type I fibers relative to type II fibers was lower in 8 W versus SED in the MG, with no change in the relative proportions of type II fibers (Figure 7F). Additionally, there was an increase of type IIa relative to the other type II fibers in 8 W versus SED, in the PL only (Figure 7G). In Aged females, there was an increase in type IIx relative to type IIb fibers in the LG with 8 wk of training (Figure 7I), and an increase of type IIa relative to other type II fibers in both the MG (Figure 7J) and PL (Figure 7K). There was no change in the SOL fiber type composition in Aged females (Figure 7L) or males (Figure 7P). In 8 W versus SED Aged males, there was a higher proportion of type IIa fibers, relative to the other type II fibers, in the MG (Figure 7N) and PL (Figure 7O), and an additional shift from type IIb to type IIx fibers in the PL (Figure 7O); there were no fiber type distribution changes in the LG (Figure 7M).

**Figure 8.**
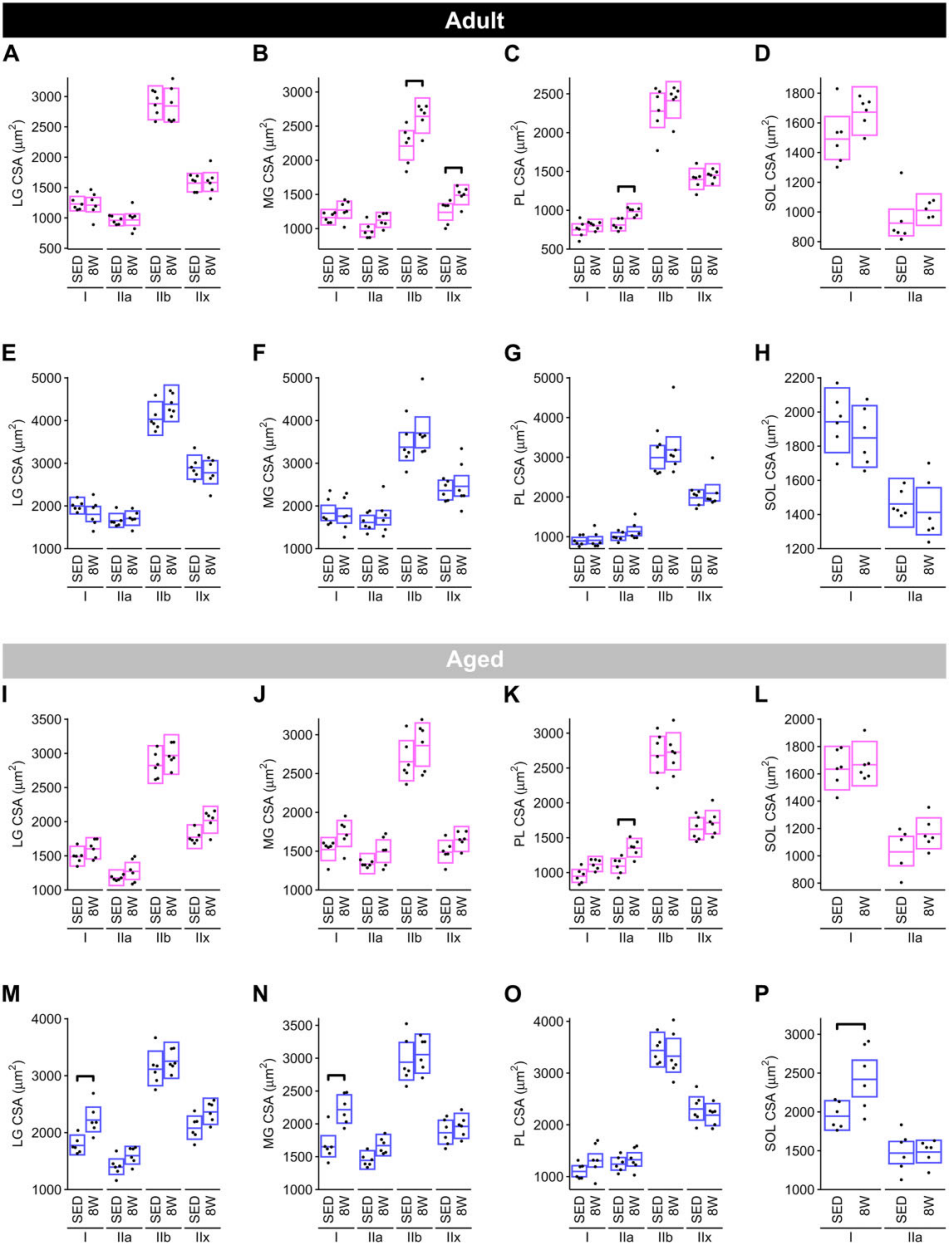
Fiber-type-specific CSA. Mean CSA of each fiber type for the LG, MG, PL, and SOL muscles from Adult females (**A**)**–**(**D**), Adult males (**E**)**–**(**H**), Aged females (**I**)**–**(**L**), and Aged males (**M**)**–**(**P**). Boxes are 95% confidence intervals for the mean CSA of each group. For each muscle and fiber type, the 8 W trained group was compared to SED, and *P*-values were Holm-adjusted across all fiber types for a given combination of age, sex, and muscle. Brackets indicate a statistically significant difference between groups (Holm *P* < .05).

*Fiber type-specific CSA*. In the Adult female rats, the mean CSA of type IIx and type IIb fibers in the MG (Figure 8B) and type IIa fibers in the PL (Figure 8C) increased significantly (MG IIx and IIb: +20%; PL IIa: +21%) in 8 W relative to SED. There were no changes in the CSA of fibers in the LG (Figure 8A) or SOL (Figure 8D) of females. In Adult males, there was no effect of treadmill training on fiber type-specific CSA in any of the four skeletal muscles (Figure 8E–H). In Aged female rats, the mean CSA of the type IIa fibers in the PL (Figure 8K) increased by + 20% at 8 W relative to SED, with no changes in the CSA of fibers in the LG, MG, or SOL (Figure 8M, Figure 8N, and Figure 8L, respectively). In Aged males, the mean CSA of type I fibers increased at 8 W relative to SED in the LG (+25%; Figure 8M), MG (+34%; Figure 8N), and SOL (+24%; (Figure 8P), but not the PL (Figure 8O).

#### Mean Muscle Fiber CSA

In Adult rats, no differences were observed in the mean muscle fiber CSA of the 8 W and SED groups of any muscles of female (Figure S8A) or male rats (Figure S8B). In Aged female rats, no changes in mean muscle fiber CSA were observed between the 8 W and SED (Figure S8C). In Aged males, 8 W SOL mean muscle fiber CSA was higher (+23%), as compared to the SED group (Figure S8D). In contrast, there were no differences in mean muscle fiber CSA in LG, MG, or PL (Figure S8D).

#### Capillary Contacts Per Muscle Fiber

Treadmill training for 8 wk had no effect on the mean number of capillary contacts per fiber in any muscle in Adult (Females: Figure S9A; males: Figure S9B) or Aged rats (Females: Figure S9C; males: Figure S9D).

#### Muscle CS Activity

**Figure 9.**
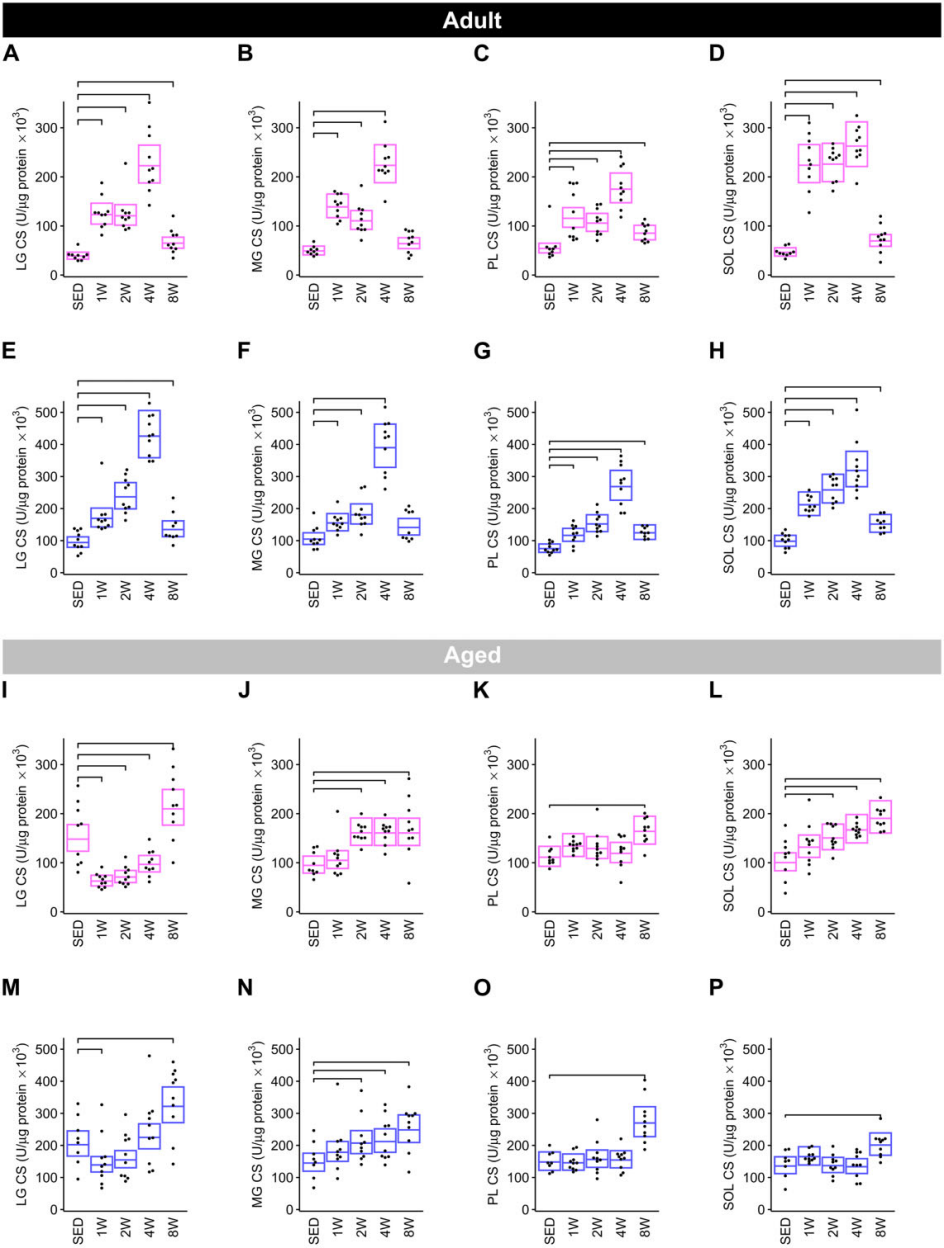
Citrate synthase activity by muscle. Citrate synthase activity in the LG, MG, PL, and SOL muscles of Adult females (**A**)**–**(**D**), Adult males (**E**)**–**(**H**), Aged females (**I**)**–**(**L**), and Aged males (**M**)**–**(**P**). Each trained group was compared against the SED group using the Dunnett test. Brackets indicate a significant change in CS from SED to trained (Dunnett *P* < .05).

*Adult group*. In Adult female rats, CS activity increased in all muscles with progressive endurance training (Figure 9A–D) and was most pronounced at 4 W. At 8 W in Adult females, CS activity increased relative to SED in the LG (+67%; Figure 9A), PL (+58%; Figure 9C), and SOL (+50%; Figure 9D), but not the MG (Figure 9B). In females, the mean fold-increase in CS activity between SED rats and apex levels in the 4 W group was—LG: +5.73, MG: +4.55, PL: +3.24, and SOL: +5.67. The impact of training on CS activity followed a similar temporal pattern in males, with significant increases in CS activity in the LG (Figure 9E), PL (Figure 9G), and SOL (Figure 9H) in all training groups, whilst in the MG the increase was evident in 1, 2, and 4 W only (Figure 9F). In the LG, PL, and SOL, the average increase in CS activity in the 8 W group relative to SED was + 43%, +65%, and + 54%, respectively (Figure 9E and G–H). In males, average fold-increases in CS activity between the SED and peak activity at 4 W was—LG: +4.52, MG: +3.72, PL: +3.56, and SOL: +3.25.

*Aged group*. In Aged female rats, CS activity was elevated by 42%–90% in all muscle groups at 8 W relative to SED (Figure 9I–L). The MG (Figure 9J) and SOL (Figure 9L) of females displayed increases in CS activity in the 2, 4, and 8 W groups relative to SED (SOL: +50% at 2 W, +66% at 4 W, +90% at 8 W; MG: +70% at all timepoints), while CS activity in the PL (Figure 9K) was only elevated at 8 W (+47%). Interestingly, in the LG of females (Figure 9I), mean CS activity was lower in the 1, 2, and 4 W groups (−58%, −52%, and −35%, respectively), but was higher at 8 W (+42%) (Figure 7I). In Aged males, temporal changes in LG CS activity were similar to females, decreasing significantly in the 1 W group (−31%), followed by an increase at 8 W (+59%) (Figure 9M). In the MG, CS activity was higher compared to SED in the 2 W (+43%), 4 W (+46%), and 8 W (+71%) groups (Figure 9N). In the PL (Figure 9O) and SOL (Figure 9P) of males, increases in CS activity were statistically different from SED in the 8 W group, only (+82% and + 48%, respectively).

#### Muscle Glycogen

**Figure 10.**
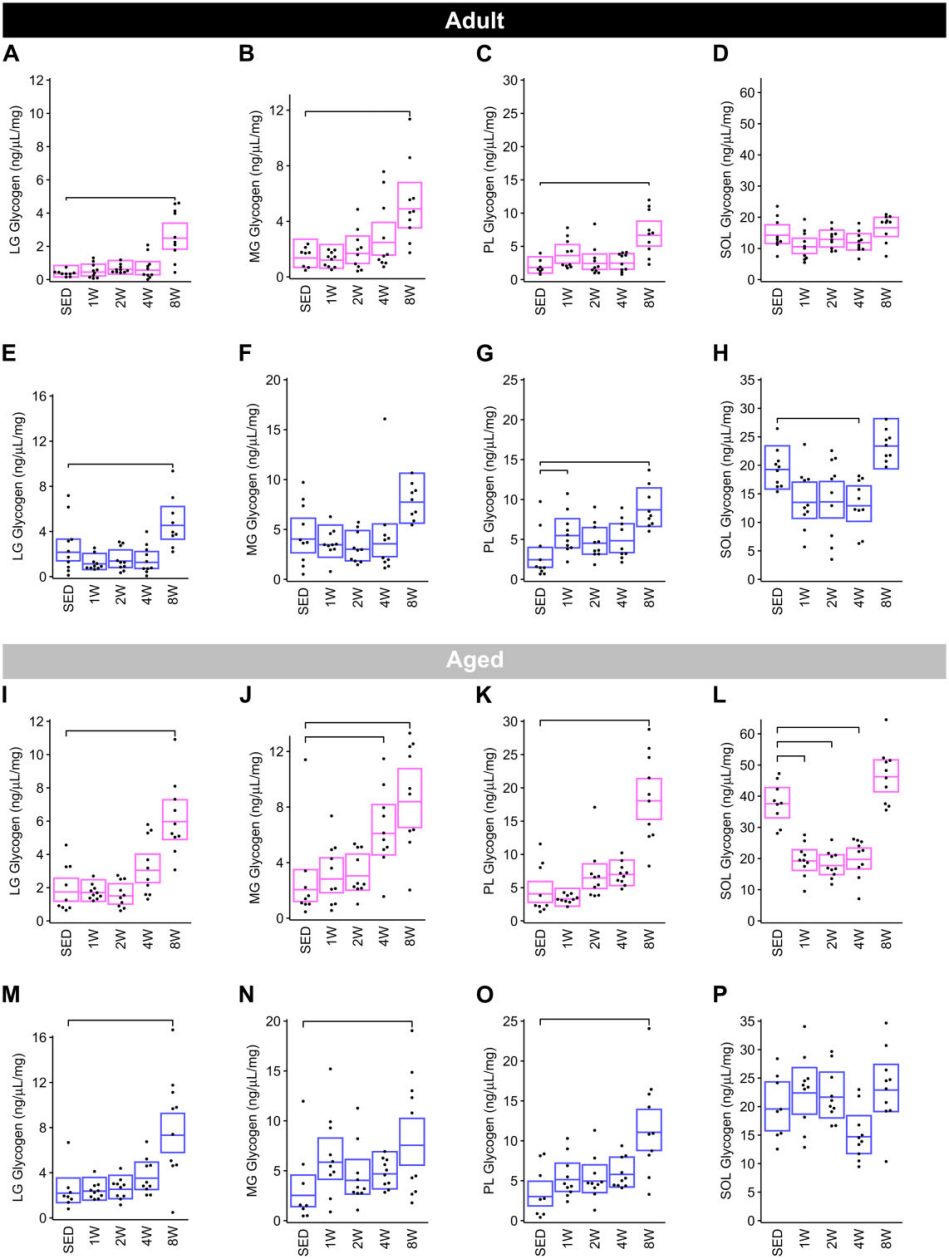
Glycogen by muscle. Glycogen concentration in the LG, MG, PL, and SOL muscles of Adult females (**A**)**–**(**D**), Adult males (**E**)**–**(**H**), Aged females (**I**)**–**(**L**), and Aged males (**M**)**–**(**P**). Each trained group was compared against the SED group using the Dunnett test. Brackets indicate a significant change in glycogen from SED to trained (Dunnett *P* < .05).

*Adult group*. In female rats, muscle glycogen content was significantly higher in the LG (Figure 10A), MG (Figure 10B), and PL (Figure 10C) of 8 W trained groups relative to SED (mean fold-changes of + 6.89, +3.60, and + 3.63, respectively). Glycogen content was not affected by training in the SOL (Figure 10AD), or at other timepoints in the other muscles (Figure 10A–C), in female rats. Glycogen content in males displayed similar training responses as females, with a few exceptions (Figure 10E–H). Similar to females, glycogen content was elevated in the LG (Figure 10E) and PL (Figure 10G) at 8 W by + 2.10-fold and + 3.55-fold, respectively, though there was no change in MG (Figure 10F) glycogen with training. In the PL (Figure 10G) there also was an early increase at 1 W (2.23-fold), and in the SOL (Figure 10H) a 33% decrease in the mean glycogen at 4 W only.

*Aged group*. In the Aged females, glycogen content increased in the 8 W group relative to SED in the LG (+3.43-fold; Figure 10I), MG (+4.08-fold; Figure 10J), and PL (+4.44-fold; Figure 10K). The MG in females also displayed an increase in glycogen content at 4 W by + 2.97-fold (Figure 10J). In the SOL, no increases were observed, with glycogen content being significantly lower in the 1, 2, and 4 W groups, as compared to SED (Figure 10L). In males, glycogen concentration was higher in 8 W, as compared to SED, in the LG (Figure 10M), MG (Figure 10N), and PL (Figure 10P), with fold-increases of + 3.34, +2.98, and + 3.67, respectively (Figure 10M–P).

### Plasma Clinical Analytes

To understand metabolic changes induced by training, we profiled a set of plasma hormones (insulin, glucagon, corticosterone, and leptin) and metabolites (glucose, lactate, NEFA, glycerol, and total ketones) that are key indicators of metabolic homeostasis.

**Figure 11.**
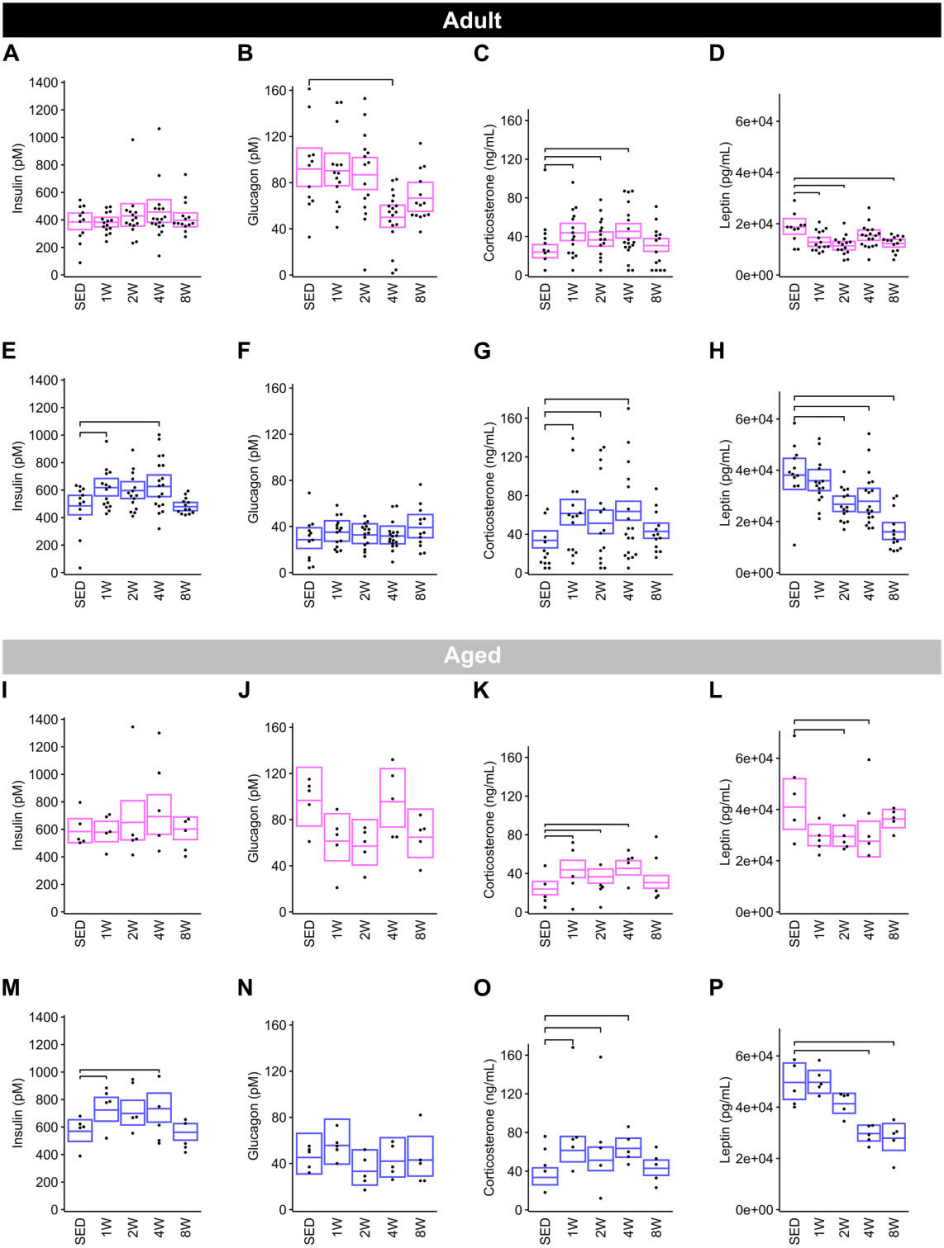
Systemic hormones. Levels of plasma insulin, glucagon, corticosterone, and leptin in Adult females (**A**)**–**(**D**), Adult males (**E**)**–**(**H**), Aged females (**I**)**–**(**L**), and Aged males (**M**)**–**(**P**). Each trained group was compared against the SED group using the Dunnett test. Brackets indicate a significant change in these hormones from SED to trained (Dunnett *P* < .05). Measurements were performed in all Adult rats, and only in the -omics cohort of Aged rats.

*Adult group hormones*. In females, plasma insulin was not impacted by training, though glucagon concentration was 46% lower in the 4 W group relative to SED (Figure 11A–B). In males, plasma insulin was elevated in the 1 and 4 W groups, with no significant differences between 8-wk-trained and SED rats (Figure 11E). Unlike females, glucagon was unaffected by training in males (Figure 11F). Corticosterone was elevated in the 1 and 2 W females, peaked at 4 W (90% increase from SED), and then returned to SED levels by 8 W (Figure 11C). A similar pattern in plasma corticosterone was observed in males, with increases in early training timepoints that were not observed by 8 W (Figure 11G). With training, plasma leptin levels decreased at 1, 2, and 8 W in females (−32%, −39%, and −34%, respectively) (Figure 11D). In males, leptin decreased at 2, 4, and 8 W relative to SED (−30%, −27%, and −58% respectively), with the greatest reduction in 8 W males (Figure 11H).

**Figure 12.**
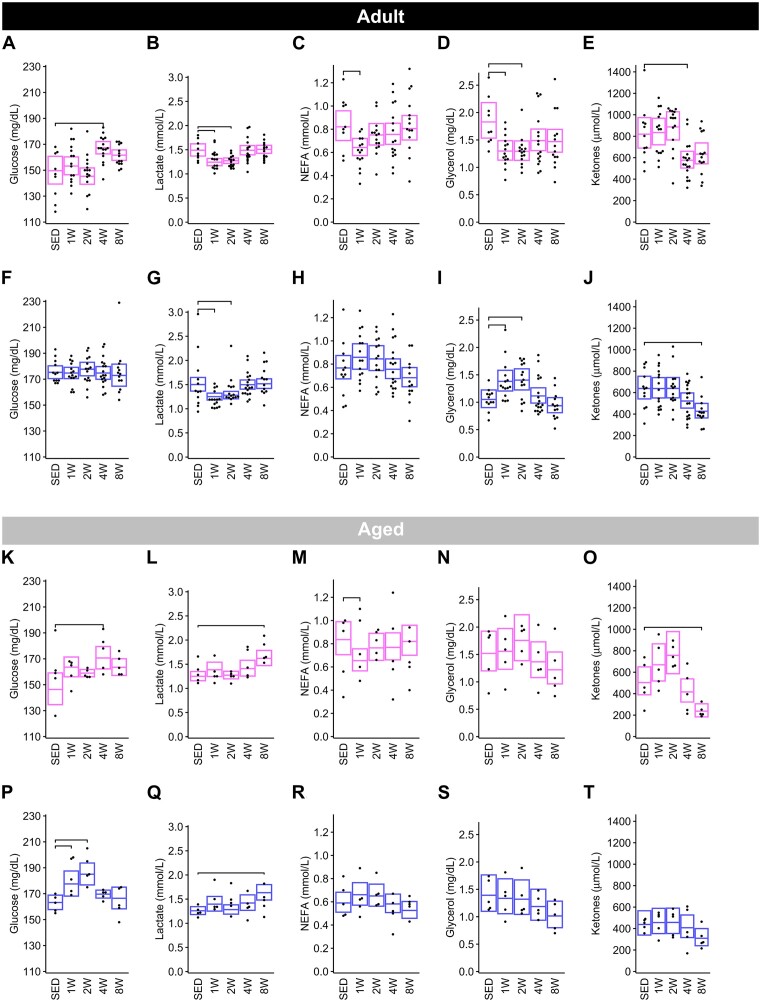
Clinical metabolites. Levels of plasma glucose, lactate, NEFA, glycerol, and total ketones in Adult females (**A**)**–**(**E**), Adult males (**F**)**–**(**J**), Aged females (**K**)**–**(**O**), and Aged males (**P**)**–**(**T**). Each trained group was compared against the SED group using the Dunnett test. Brackets indicate a significant change in these metabolites from SED to trained (Dunnett *P* < .05). Measurements were performed in all Adult rats, and only in the -omics cohort of Aged rats.

*Adult group plasma metabolites*. In Adult females, several plasma metabolites displayed early training responses that were attenuated with prolonged training (Figure 12A–E). Namely, lactate and glycerol decreased in the 1 and 2 W groups relative to SED (lactate 1W: −17%, 2W: −15%; glycerol 1 W and 2W: −29%), and NEFA decreased by 22% at 1 W. Glucose and total ketone bodies were the only metabolites to respond at 4 wk of exercise training in females, with glucose increasing by ∼12% and ketones decreasing by ∼30% before mostly returning to SED levels by 8 W. In Adult males (Figure 12F–J), similar to Adult females, plasma glucose levels were not impacted by training, while plasma lactate decreased at 1 and 2 W of training (−17% and −15%, respectively). Interestingly, training had opposite impacts on glycerol concentrations in males—causing an increase of 31% with 1 W and 34% with 2 W of training before returning to SED levels at the later timepoints (Figure 12D, I). Finally, total ketone bodies displayed a similar temporal pattern in both sexes, decreasing significantly in 8 W males (−33%) (Figure 12J).

*Aged group hormones*. The impact of training on plasma hormones in Aged rats followed similar temporal patterns as that of Adult rats for all analytes with the exception of glucagon in Aged females, which did not change with training (Figure 11J). Plasma insulin was unaffected by training in females (Figure 11I) and increased at 1 and 4 W in males (+27% and + 29%, respectively) (Figure 11M). As in Adult animals of both sexes, plasma corticosterone was elevated at 1, 2, and 4 W training timepoints (+83, +53%, and + 90%, respectively) and then returned to SED levels at 8 W (Figure 11K). Leptin appeared to decrease with training, though the response was attenuated in Aged females, with marginal decreases that were only significant in the 2 W (−28%) and 4 W (−33%) groups (Figure 11L). In Aged males, the leptin training response also appeared attenuated relative to Adult males, with plot trajectories suggesting a decrease beginning at 2 wk, but was not significant until 4 W (−40%) of training (Figure 11P); an additional 4 wk of training did not appear to lower leptin much further (−44% from SED to 8 W).

*Aged group plasma metabolites*. In Aged females (Figure 12K–O), glucose and NEFA responded similarly to training as they did in Adult females, where glucose increased at 4 W (17%) and NEFA decreased at 1 W (−22%). In males, glucose increased by 9% at 1 W and peaked at 2 W (13%) (Figure 12P) relative to SED, whereas plasma NEFA did not significantly change with training. In comparison to Adult males and females, where lactate decreased at 1 and 2 W of training (Figure 12B and G), lactate levels in Aged rats of both sexes trended upwards, increasing ∼30% in 8 W relative to SED groups (Figure 12L and Q). Also, unlike their younger Adult counterparts, glycerol did not appear to respond to training, though the plots suggest it may have begun to decrease at later timepoints (Figure 12N and S). Finally, while ketones displayed nonsignificant increases at 1 and 2 W in females, levels decreased suddenly around 4 W and continued until becoming 53% lower at 8 W relative to SED (Figure 12O). In males, ketones did not significantly change with training, although they did display a downward trend similar to Adult males (Figure 12T).

## Discussion

While the health benefits of regular endurance exercise are widely known,^52^ the separate and integrative effects of exercise training on molecular signaling across many tissues, and how this interrelates to health and disease risk, remains to be thoroughly defined. To address this knowledge gap, here we describe the endurance exercise training arm of the Preclinical Animal Study Sites (commonly referred to as PASS1B) of MoTrPAC, the primary goals of which were to, (1) develop a standardized endurance exercise protocol for the characterization of physiological adaptation to exercise and, (2) collect an expansive group of tissues/organs for the creation of a publicly accessible tissue biorepository and multi-omic analysis database. Specifically, we examined key physiological and metabolic adaptations after 1, 2, 4, or 8 wk of endurance exercise treadmill training at ∼70%–75% VO_2_max in a large cohort of male and female F344 rats.

Importantly, in relation to the goals of this work, the progressive endurance training program resulted in a robust (∼20%) improvement in cardiorespiratory fitness regardless of age or sex, with variable impacts of age and sex on other phenotypic measures. Moreover, extensive phenotypic data from 294 rats was collected and > 5600 total samples comprising 18 solid tissues and blood were collected and biobanked, making this the most expansive, publicly accessible data resource and tissue biorepository, to date, for studying temporal, multiomic, sex-specific, and age-specific responses to progressive endurance training. Indeed, the utility of this resource is exemplified by a recent landscape study by MoTrPAC, which investigated the multiomic response within and across tissues in a subset of the male and female Adult rats from PASS1B.^28^ Additionally, more focused studies leveraged this multiomics data to study the tissue-wide mitochondrial^29^ and transcription factor^27^ response to training, or sex- and training-specific responses in subcutaneous white adipose tissue.^26^

A key strength of the PASS1B resource is the expansive cohort size; to our knowledge, it is the first study of such magnitude to document these progressive changes in male and female rats of two age groups. Up to now, most rodent and human molecular profiling studies have been limited to studying a single time point, sex, and/or age group, which limits the broader application and interpretation of the findings. Notably, studying the progressive response to endurance training permits integration of physiological and -omic adaptations across tissues, thereby offering the opportunity to reveal novel pathways key in tissue remodeling. An additional unique aspect of PASS1B is the highly controlled experimental design, which increases the translatability and reproducibility of the work. For example, to aid reproducibility training and tissue collection occurred over a restricted time period and at the same time of day, which was during their normally active dark phase. To accomplish tissue collection over a narrow time period, and minimize potential circadian effects, we staggered the rat training schedule. Such an approach likely resulted in female rats being staggered throughout their 5 d estrous cycle, which would limit biasing to one phase of the estrous cycle. Our experimental design also ensured that all animals received the same degree of human handling, and environmental conditions (eg, bedding, feed, and ambient conditions) that were consistent upon animal arrival and throughout the study. Our treadmill protocol was carefully chosen to allow progressive training of rats at standardized workloads.

While common endurance exercise-based interventions in rats include voluntary wheel running, swimming, and treadmill running, the latter was chosen for several reasons. First, compared to swimming, which primarily employs the flexor muscles, running is a whole-body exercise modality that uses hindlimb flexor and extensor muscles.^53^ Second, the principle of progressive overload forms the foundation of a successful exercise intervention and treadmill training allows the exercise stimulus to “progress” in a controlled manner, thereby inducing an adaptive response.^54^ Treadmill training is also continuous—mimicking programmed exercise in humans—while wheel running is intermittent in nature. While there are advantages and disadvantages to all exercise modalities,^55^ the consensus of the consortium was that treadmill training was the best mode of endurance exercise to meet the goals of MoTrPAC, which included the potential to overlay and translate the preclinical animal studies in F344 rats (be it after acute exercise or exercise training) to that of the human arm of MoTrPAC.

A key adaptive response to endurance exercise training is an increase in cardiorespiratory fitness or VO_2_max.^56^,^57^ Here, our training regime followed the seminal rat training study of Wisløff et al.,^30^ with modifications to a lower targeted continuous moderate-to-high intensity of 70%–75% VO_2_max. This intensity not only provides translational relevance to humans,^19^,^30^,^58^,^59^ it also assimilates with the training protocol and target intensity of the human MoTrPAC studies.^18^ Moreover, targeting this intensity (at a minimum) is important for treadmill training in Aged rats, as training at lower intensities does not elicit equitable improvements in VO_2_max when compared to young counterparts.^60^,^61^ Therefore, we chose a treadmill speed and grade to elicit similar relative oxygen consumption in Adult and Aged rats^60^,^61^ and humans.^59^,^62^ In line with the work of Wisløff,^30^ we observed a robust improvement in absolute and relative VO_2_max after 4 wk of training, which continued to increase with 8 wk of training, in Adult, male and female rats, pairing with previous studies in various strains of rats.^30^,^63^,^60^,^64–66^ Consistent with the well-defined linear relationship between VO_2_ and workload,^30^ maximal run speed (MRS) increased in male and female Adult rats progressively between 4 and 8 wk of training. Changes in VO_2_max were only measured at the 8-wk timepoint in Aged rats and also showed substantial improvements. While VO_2_max was not measured at the 4-wk timepoint in Aged rats, MRS increased at 4 wk followed by further improvements at 8 wk indicative of progressive improvements in cardiorespiratory fitness in Aged animals. Such observations are consistent with studies in F344,^60^,^64^ other rat strains,^65^,^66^ and humans,^59^,^67–69^ which demonstrate a 1%–31% increase in VO_2_max in response to training at a similar continuous intensity (60%–80% VO_2_max). It is notable to mention that interval treadmill training is capable of inducing more robust improvements in VO_2_max in rats^30^,^63^ and humans.^70^ While baseline and adaptability in VO_2_max to training can differ between inbred strains of rats^66^ and amongst outbred rats,^71^ observations of similar percentage improvements between rats and humans substantiate our training protocol utility and reproducibility. Interestingly, when looking at individual training responses in VO_2_max, animals with a higher baseline VO_2_max tended to have a lower improvement in VO_2_max with training as compared to those with a lower baseline VO_2_max. This is likely because each group of animals, regardless of their baseline VO_2_max, trained at the same workload (ie, 70%–75% of the average VO_2_max for the cohort); as such, those with a lower baseline VO_2_max were likely training at a higher relative percentage of VO_2_max, and thus might be expected to have a greater adaptive response. Overall, these data validate that the training protocol developed and implemented in PASS1B promotes similar cardiorespiratory adaptations in male and female Adult and Aged rats—warranting additional investigation of systemic responses to progressive endurance training.

Endurance exercise training can profoundly affect body composition—especially body fat.^72–74^ Here, training-induced changes in body composition were influenced by both age and sex. Over the 8-wk training period, fat mass decreased in both Adult and Aged males, whilst in females it only decreased in Aged, but not Adult rats. While Adult females did not lose fat mass with training, it should be noted that exercise training prevented the Adult females from gaining fat mass, as occurred in the age-matched SED rats. Sexual dimorphism in endurance training-induced fat loss is observed in weanling rats,^75^ with females potentially displaying attenuated fat loss relative to males. While mixed in the literature in humans,^76^,^77^ endurance training appears to promote greater degrees of fat loss in postmenopausal versus premenopausal females.^78–81^ Mechanisms of attenuated fat loss in female rodents and humans may be attributable to increased compensatory food consumption^82^,^83^ and other evolutionary conserved molecular mechanisms to maintain reproductive fitness in females.^26^,^75^,^84^ Notably, while the underlying reason for changes in body and fat mass were not investigated in this study (all animals had *ad libitum* access to food and we did not measure food intake or 24 h energy expenditure), recent multiomic work by MoTrPAC in the subcutaneous WAT (scWAT) of a subset of these Adults rats identified candidate molecules and pathways regulating sexually dimorphic responses to exercise training.^26^ Interestingly, despite attenuated fat loss in Adult female rats, all groups decreased plasma leptin levels following training, which is suggestive of adipose tissue remodeling toward a healthier phenotype and/or a decrease in visceral fat mass.^85^ Both Adult and Aged males displayed greater reductions in plasma leptin, pairing with changes in total fat mass and increase in glycerol levels in Adult males at 1 and 2 wk, indicative of lipolysis in the early training response. It is important to note that training-associated reductions in total body mass were accompanied by decreases in total lean mass in Aged, but not Adult male rats, and Aged female rats. Despite these reductions, the relative percentage of lean to total fat mass increased in Aged male, but not Aged female rats. An important point when interpreting these body composition changes is which body compartments the whole-body NMR is measuring. For example, lean (body) mass measurement is an assessment of all lean tissues, including skeletal muscle, liver, lungs, kidneys and heart; it does not include bone minerals, fat, and substances which do not contribute to the NMR signal, such as hair and claws. Thus, decreases (or changes in general) in total lean mass are not necessarily reflective of a decrease in skeletal muscle mass, but could be due to changes in mass in other tissue types. To this point, while there was a robust decrease in lean mass in Aged rats, terminal masses of LG, MG, PL, and SOL were similar between SED rats and the trained groups, and were even increased in 8 W Aged females, suggesting that the lower lean body mass in Aged trained groups may be due to reduced mass of other lean tissue compartments, not skeletal muscle mass. Thus, direct measures of skeletal muscle mass(es), where possible, can be helpful when interpreting body composition data, such as that provided by whole-body NMR.

Glucocorticoids play an important role in the adaptation to a variety of homeostatic stressors that perturb homeostasis, including exercise. All training groups displayed an increase in plasma corticosterone with progressive endurance training (weeks 1–4); levels attenuated at 8 wk following a 2-wk plateau in training intensity and volume. Similar to our findings, during the initial weeks of chronic exercise training (up to 4 wk), plasma corticosterone concentrations have been reported to be higher in the rested and post acute exercise or restraint state and to decrease during subsequent training weeks as chronic central adaptations occur.^86^,^87^ Potential implications for this increase include effects on metabolism through actions on multiple organs including the liver, adrenals, brain, skeletal muscle, and white adipose tissue.^88^

Given that skeletal muscle metabolic adaptations are essential for whole-body improvements in aerobic fitness with endurance training,^89^ we assessed changes in CS, capillarization, and glycogen in four hindlimb muscles. As CS catalyzes the first step of the Krebs cycle, its activity is commonly used as a marker of skeletal muscle oxidative capacity. To this point, our training protocol resulted in a robust increase in CS activity in both Adult and Aged rats, regardless of sex, and in multiple muscles that were tested. Overall the changes we observed are consistent with previous training studies that measured CS activity^65^ or SDH activity^60^ in F344 rats of similar ages. Nevertheless, the temporal dynamics of CS activity did differ between Adult and Aged rats. In Adult trained rats, CS activity peaked at 4 wk, and then decreased by 8 wk, albeit to levels higher than SED rats. Given the training protocol in weeks 7 and 8 was designed to be at a steady state, the adaptations in Adult rats may reflect a plateau in mitochondrial adaptations that occur in the absence of increased intensity, as originally proposed by Dudley.^58^ Importantly, this decrease did not impact the increase in VO_2_max after 8 wk of training in Adult rats. Conversely, CS activity peaked at 8 wk in Aged rats, which may be reflective of differences in the rate of change in training intensity between the Adult and Aged rats between weeks. Alternatively, the continued increase in CS activity despite a plateau in training volume and intensity in Aged rats, has also been reported in humans,^62^ where changes in vastus lateralis respiratory capacity following endurance training at 70% VO_2_max were observed to be higher in older relative to young subjects despite similar improvements in aerobic fitness.^59^ Nevertheless, it is important to not overinterpret changes in mitochondrial content based on one mitochondrial enzyme. To this point, recent multi-omic analysis of skeletal muscle (and the other tissues collected from this study in Adult rats) demonstrated a robust improvement in multiple markers of oxidative metabolism and mitochondrial capacity that were sustained through 8 wk of training.^28^,^29^

Chronic endurance exercise has been shown to induce angiogenesis and increase capillarization in both human and rodent skeletal muscle.^90^,^91^ Changes in capillarity have been measured by a number of methods including: capillary density, capillary-to-fiber ratio, and capillary contacts. We measured the mean capillary contacts of fibers in four muscles of variable fiber type composition (SOL, PL, MG, and LG) and activity patterns during the moderate intensity exercise training. We found no significant increase in mean capillary contacts in the four muscles studied following 8-wk of treadmill training. The lack of an increase could be related to many factors including age, intensity of training, and duration of training. Our training program was at a moderate intensity for a duration of 8-wk, with the last 2 wk maintained at steady state (constant speed, incline and duration). Many studies that have observed an increase in capillarity have been at a higher intensity and for a longer duration (10–12 wk).^92^,^93^ It should be noted that our training protocol produced minimal changes in fiber CSA. While we did not find an increase in capillarity as measured by capillary contacts, we did observe that the SOL, a predominantly slow, oxidative muscle, had the highest mean capillary contacts at both ages and in both sexes. Interestingly, there was a general trend for males to have greater mean capillary contacts in all muscles compared to females.

Muscle glycogen levels are also indicative of skeletal muscle training adaptations, in part due to greater muscle GLUT4 abundance^94^ and sarcolemma translocation following acute exercise,^95^ and elevated fatty acid oxidation that occurs with training resulting in glycogen sparing.^96^ We observed increases in muscle glycogen content in all training groups at 8 wk in all muscles except for the type I fiber-dominant SOL muscle. While several human studies cite sex and age-specific differences in muscle glycogen content and glucose kinetics following training,^59^,^97^ such differences may be impacted by timing of sampling (eg, sample collection < 48 h after the last bout of exercise or before a plateau in training intensity) and/or reduced sample size in human trials. In our study, Aged animals displayed overall higher concentrations of muscle glycogen, which may be reflective of differences in muscle fiber type distribution, substrate preference during exercise, and/or functional capacity with aging.^98^,^99^ Integration of multiomic assays performed on these rats, will help identify molecular regulators contributing to age- and sex-specific differences in skeletal muscle metabolic adaptations to training.

The effect of endurance training on the fiber type composition of a muscle is dependent on the muscle type and the intensity and duration of the exercise training.^60^,^100–103^ Classification of muscle fibers based on MHC expression yields four primary fiber types in rodent limb muscle (I, IIa, IIx, and IIb) and three primary fiber types in human limb muscles (I, IIa, and IIx).^104^ In humans, endurance training has been shown to promote a shift in MHC expression from IIx toward IIa in the vastus lateralis muscle.^105^ In the current study, we found a consistent shift from type IIx/IIb to type IIa in the PL muscle of both Adult and Aged rats, regardless of sex. A shift toward more type IIa fibers was also observed in the MG and LG of Aged but not Adult rats. The greater fiber type shifts in the Aged MG/LG compared to the Adult MG/LG may reflect increased recruitment of these muscles in the Aged rats during the treadmill running. Collectively, the fiber type shifts we observed are consistent with what has been observed in previous rodent and human studies following endurance training of a similar intensity and volume.^106–110^ Interestingly, there was a noticeable difference in the IIx/IIb ratio in female versus male rats, regardless of age. Sexual dimorphism in the fiber type composition of jaw muscles has been reported,^111^,^112^ but to our knowledge sexual dimorphism in the mixed hindlimb muscles of rodents has not been studied in detail and the underlying reasons for this difference are unknown.

Given that skeletal muscle mass and fiber area is an important determinant of health and mortality, especially with advancing age,^2^,^4^,^6^,^113^ we also assessed changes in overall and fiber type-specific CSA. A total of 8-wk of endurance training did not significantly impact myofiber CSA in Adult male rats. Adult female rats, however, displayed fiber type-specific increases in the MG (IIb and IIx) and PL (IIa). Given Adult females were generally better runners than Adult males, differences on the impact of endurance training on fiber CSA could relate to differences in recruitment and external loading. Conversely, in Aged rats with training only males increased mean fiber CSA in the SOL, likely driven by type I fiber-specific increases in the SOL. Increased CSA of type I fibers in Aged males was also observed in the LG and MG at 8 W. Aged females did not gain body or lean mass with training, but did display an increase in PL type IIa myofiber CSA. Increased type IIa CSA in the PL is consistent with findings from a similar training protocol in 25-month-old female F344 rats.^102^ Aging is associated with muscle atrophy, especially of type II fibers in both rodents and humans.^60^,^114–117^ Atrophy of the type II fibers was apparent in the LG and MG of the Aged males relative to the Adult males. Collectively, our data supports a growing body of evidence that endurance training may attenuate age-associated selective fiber atrophy in older individuals.^118^

Since the seminal study by John Holloszy demonstrating the effects of endurance exercise training on mitochondrial mass and function in skeletal muscle,^19^ thousands of studies have investigated the salient effects of exercise on health and biology. Among these, other important works have described the effects of training duration and intensity, for example, on skeletal muscle mitochondria by muscle group and muscle type.^58^ However, a prominent limitation in advancing the field is the overt lack of investigation into the effects of progressive endurance exercise training over time, in males and females, at different ages, and across a comprehensive set of tissues/organs. Addressing this limitation, this work by the PASS1B arm of MoTrPAC details the physiological and metabolic adaptations to progressive endurance training and represents the most expansive study and tissue sample biobank of its kind available for public investigation and exploration. Ultimately, the goal of this resource is to foster integration of these data with integrative -omic data sets and further establish an independent and integrative molecular map of time-, sex-, and age-specific response to endurance exercise training to drive future research in the field.

## Supplementary Material

zqae014_Supplemental_Files

## Data Availability

All data, analysis results, and plots are available in the *MotrpacRatTrainingPhysiologyData R* package (https://motrpac.github.io/MotrpacRatTrainingPhysiologyData/; v2.0.0). This *R* package includes all code to prepare data, perform analyses, and generate plots (accessible through the “Articles” tab and described in the README file). A collated version of all data is also available on the MoTrPAC Data Hub: https://motrpac-data.org/publications/data/animal/phenotype/full-table-endurance-training. Notably, a subset of Adult animals from this cohort (*n* = 6 per experimental group and sex) have undergone extensive -omic profiling, with this publicly accessible resource being published by the MoTrPAC Study Group.^28^ Those animals used in the multi-omics analyses are identified in the collated data set.
