# Supplementary material for: Physiological Adaptations to Progressive Endurance Exercise Training in Adult and Aged Rats: Insights from the Molecular Transducers of Physical Activity Consortium (MoTrPAC)
Source: Function (Oxf). 2024 Mar 28;5(4):zqae014. doi: 10.1093/function/zqae014 (PMC11245678; doi:10.1093/function/zqae014)
Supplement: zqae014_Supplemental_Files [file zqae014_supplemental_files.zip › Supplementary_Figures_and_Legends.docx]

**
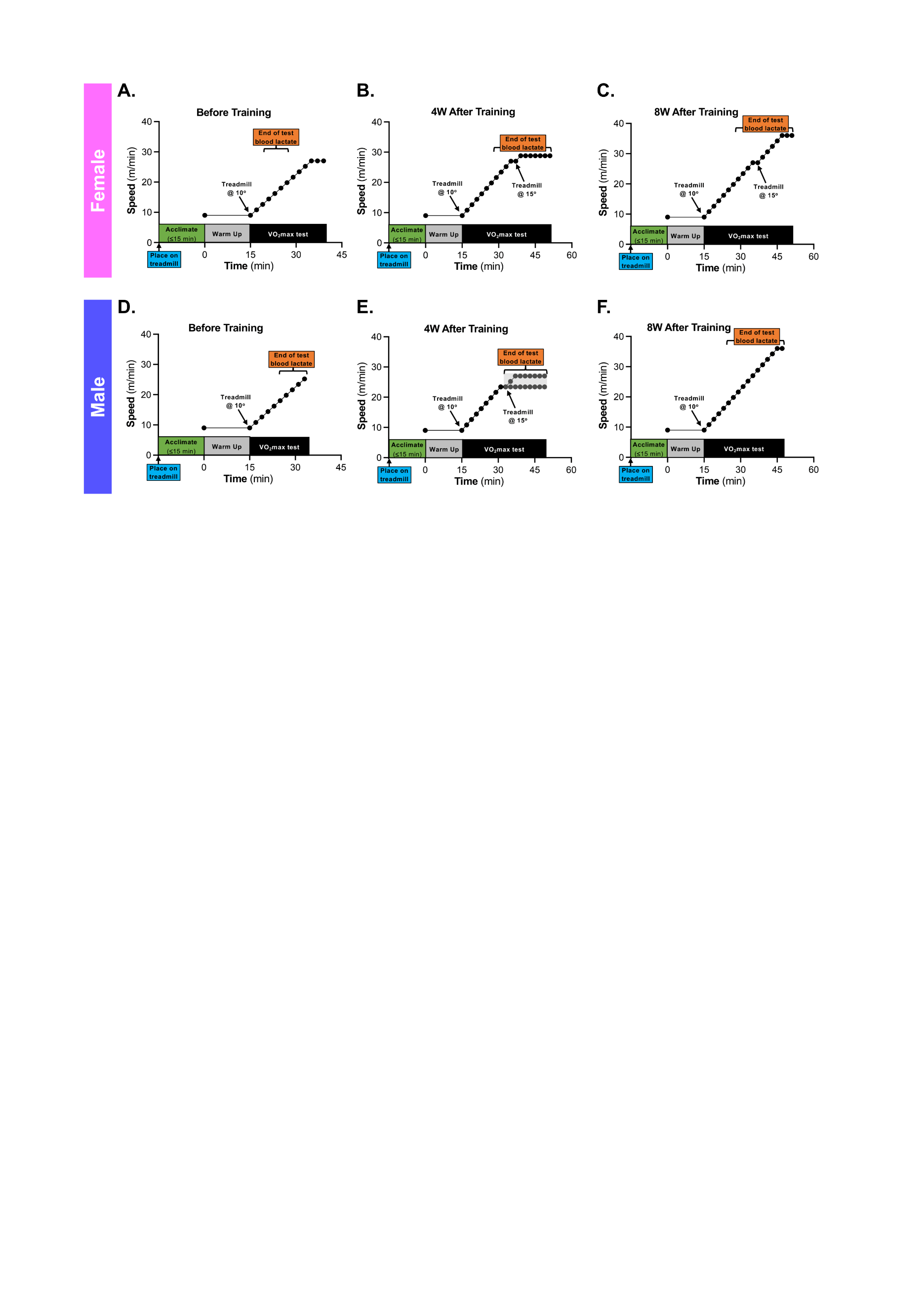
**

**Figure S1. Protocol for VO2 max testing.** Before training (**A**), after 4 weeks training (**B**), after 8 weeks training (**C**) in female rats. Before training (**D**), after 4 weeks training (**E**), after 8 weeks training (**F**) in male rats. For all testing, rats first acclimated to the treadmill, which was stationary, for up to 15 minutes. Then, the testing protocol was initiated. The first 15 min of the testing protocol was a “warm-up”, with the treadmill at 0 degrees. Then, the treadmill incline was increased to 10 degrees and speed was increased by 1.8 m/min every 2 minutes. Depending in the group, treadmill incline was increased to 15 degrees, as noted.

**
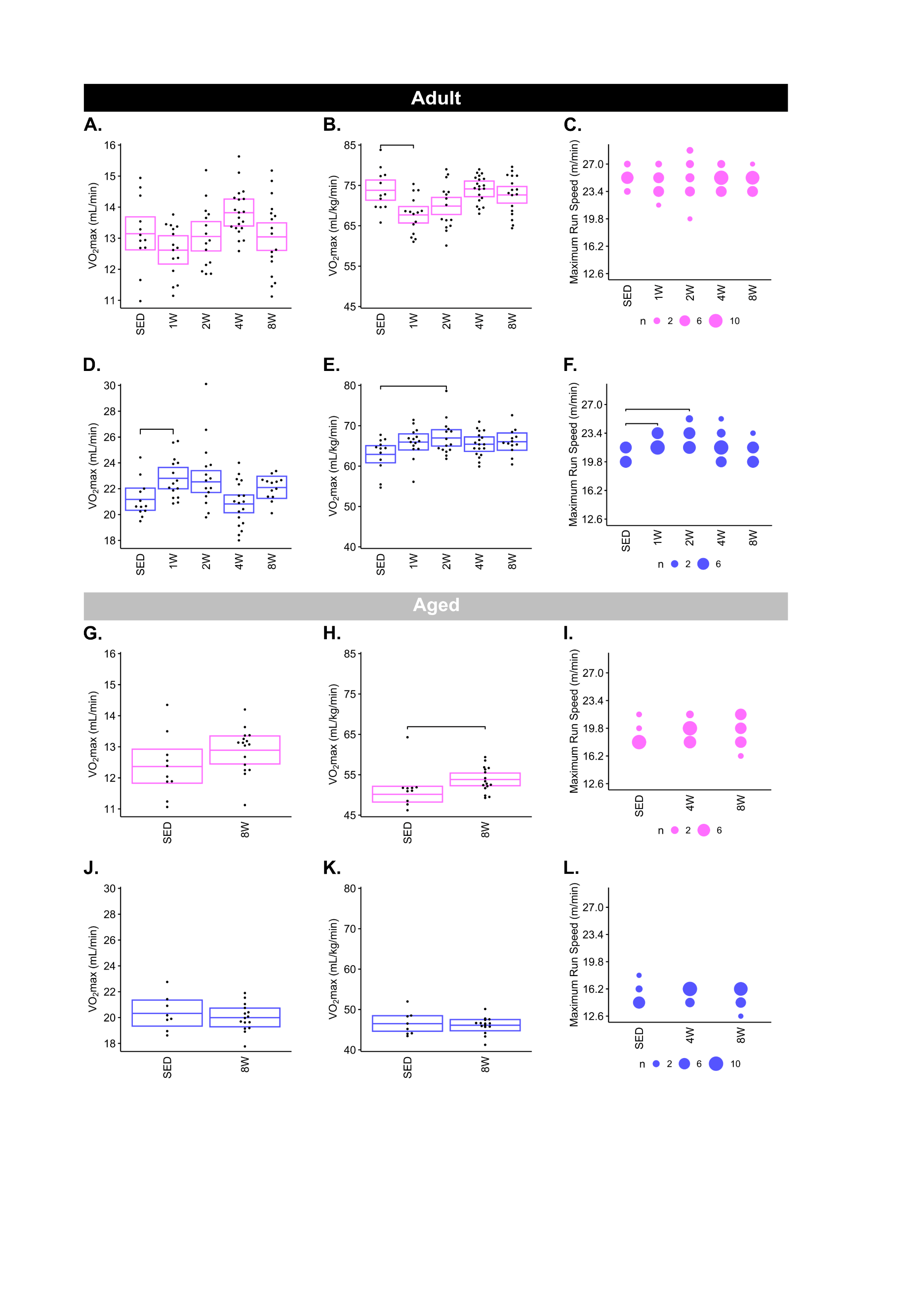
**

**Figure S2. Baseline VO_2_max and MRS.** Baseline (pre-training) measures of absolute VO_2_max, VO_2_max relative to total body mass, and maximum running speed (MRS) in Adult females (**A–C**), Adult males (**D–F**), Aged females (**G–I**), and Aged males (**J–L**). While Dunnett tests were used for absolute and relative VO_2_max to compare each trained group to SED, the Wilcoxon Rank Sum test was used instead for MRS. P-values were Holm-adjusted across each set of comparisons. Brackets indicate a statistically significant difference between groups (Dunnett p < 0.05).

**
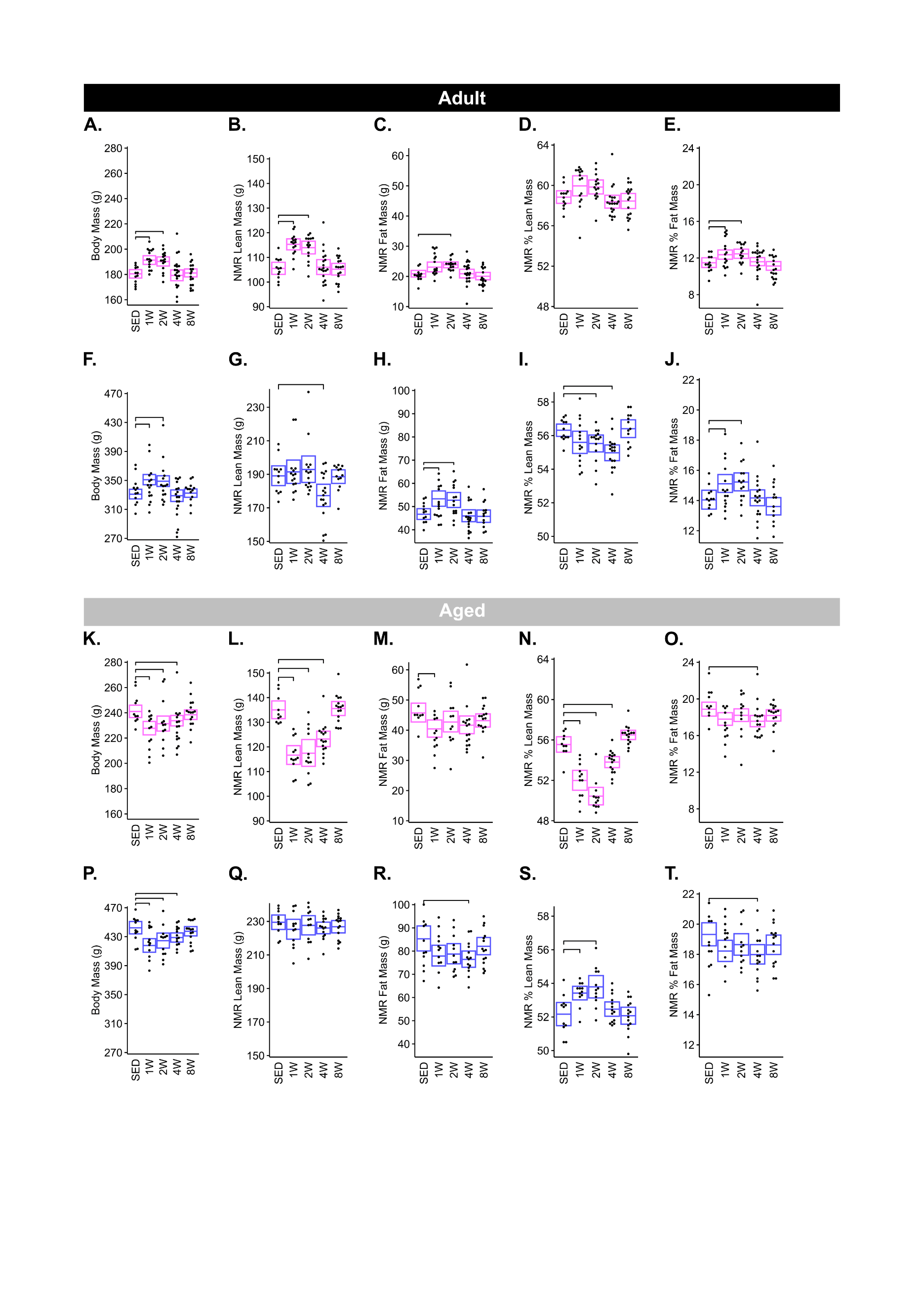
**

**Figure S3. Baseline NMR Body Composition.** Baseline (pre-training) measures of body mass, lean mass, fat mass, % lean mass, and % fat mass in Adult females (**A–E**), Adult males (**F–J**), Aged females (**K–O**), and Aged males (**P–T**). Dunnett tests were used to compare each trained group to SED. Brackets indicate a statistically significant difference between groups (Dunnett p < 0.05).

**
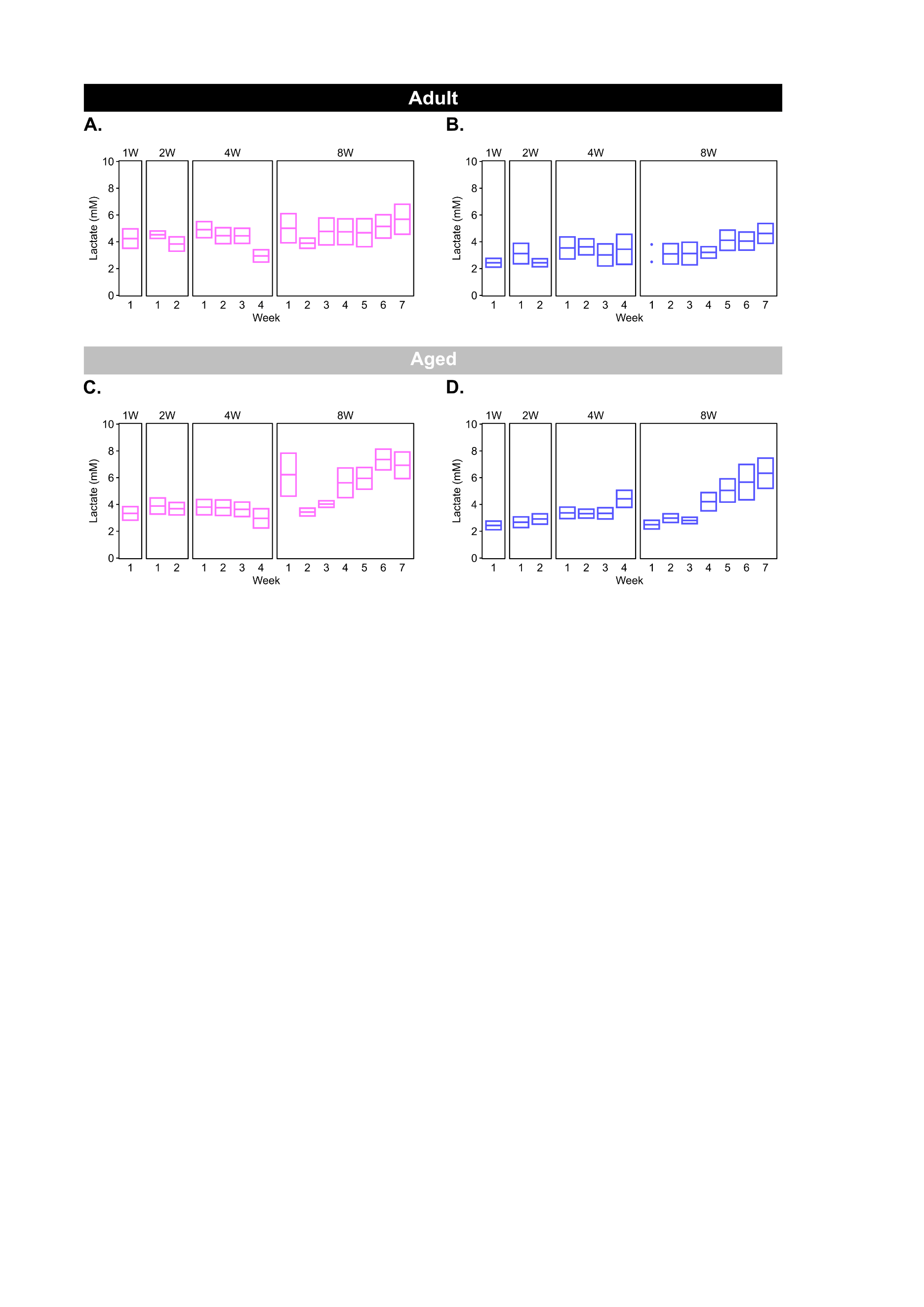
**

**Figure S4. Weekly blood lactate.** Blood lactate from Adult female (**A**), Adult male (**B**), Aged female (**C**), and Aged male (**D**) rats measured at the end of the exercise bout on the first day of each training week. Note: the week 4 values for the 4W training group animals are the end-of-week post-exercise blood lactate (day 20), since the VO_2_max testing was performed at the start of that week. Boxes are 95% confidence intervals for the mean lactate at each week, and they are colored by sex (female=pink, male=blue). Points are shown instead of confidence intervals if there are fewer than 5 observations. Statistical analyses were not performed.

**
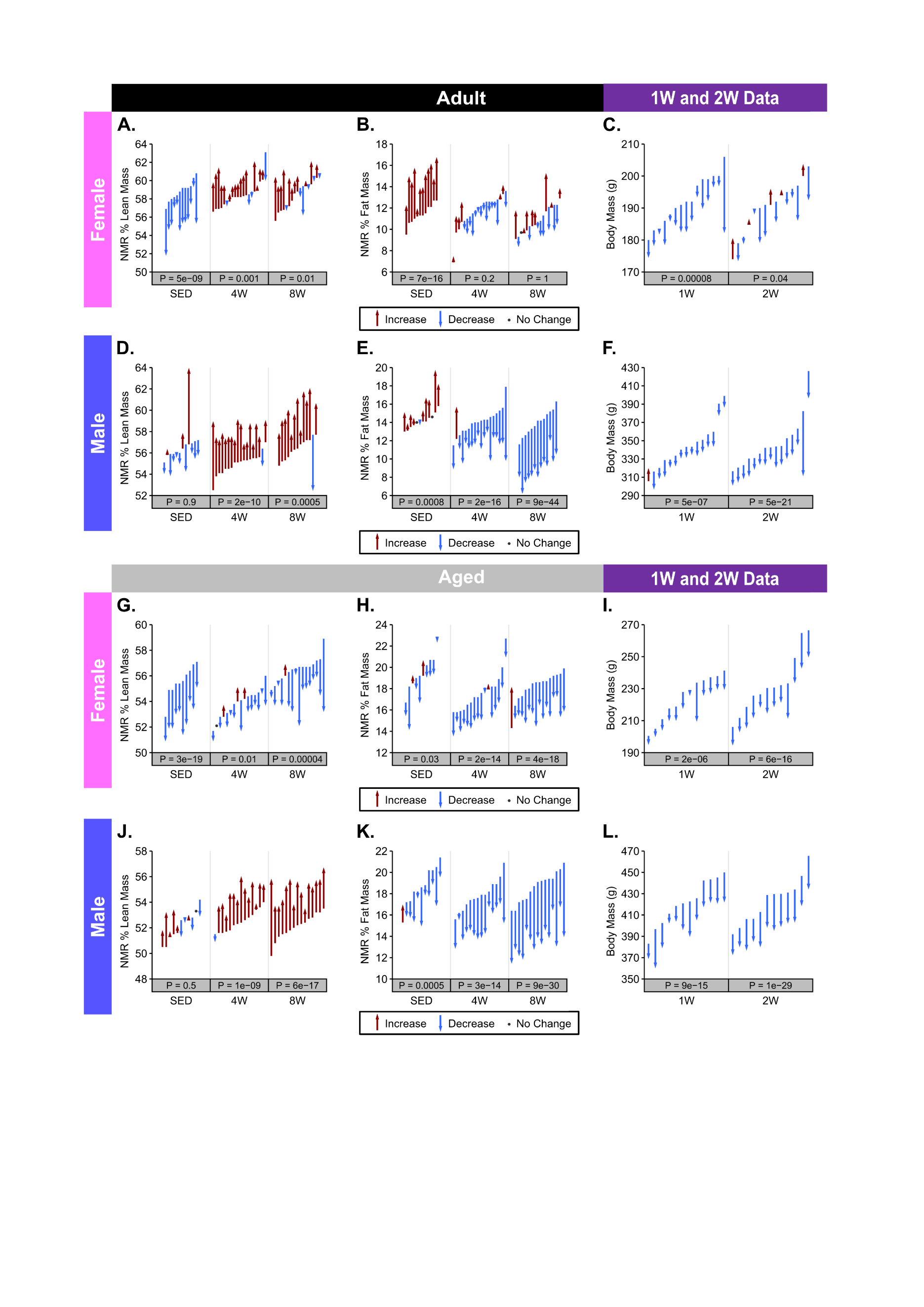
**

**Figure S5. Training NMR Body Composition.** NMR % lean mass and % fat mass and total body mass in grams (recorded on the same day as the NMR measures) from Adult females (**A–C**), Adult males (**D–F**), Aged females (**G–I**), and Aged males (**J–L**). P-values were obtained from two-sided one sample *t*-tests of the (post - pre) differences, and they were Holm adjusted within each combination of age and sex.

**
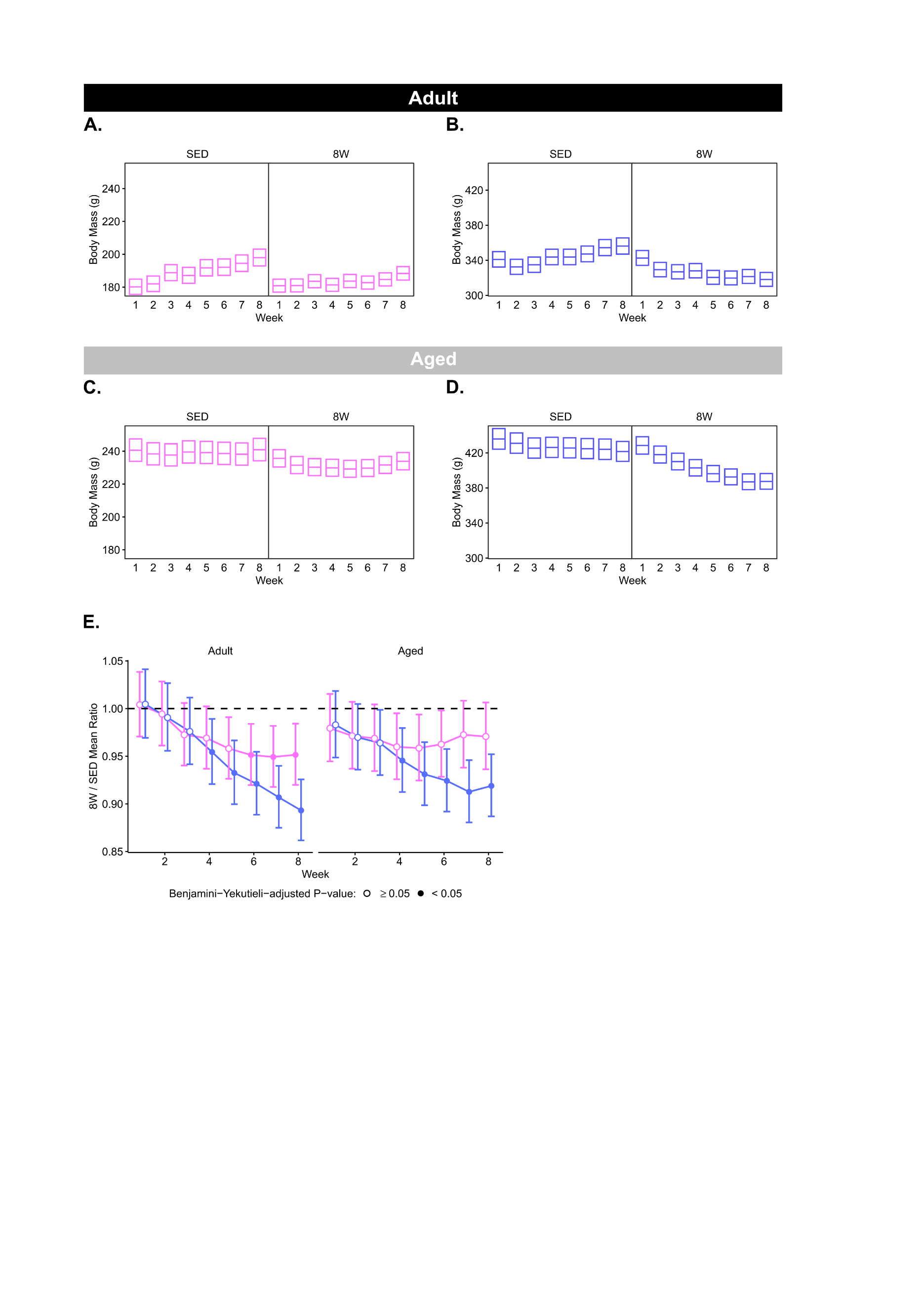
**

**Figure S6. Weekly body mass.** Body masses of Adult female (**A**), Adult male (**B**), Aged female (**C**), and Aged male (**D**) rats measured at the start of each week (prior to the onset of training that week) in the SED and 8W trained groups. Boxes are 95% confidence intervals for the mean of each group, and they are colored by sex (female=pink, male=blue). **E**) Results of statistical analysis of the GLS model with 95% confidence intervals for ratios between the means of the 8W and SED groups (“Weekly Body Mass” Methods). A solid circle for the mean indicates that the Holm-adjusted p-value was less than 0.05, while an open circle indicates that it was not.

**
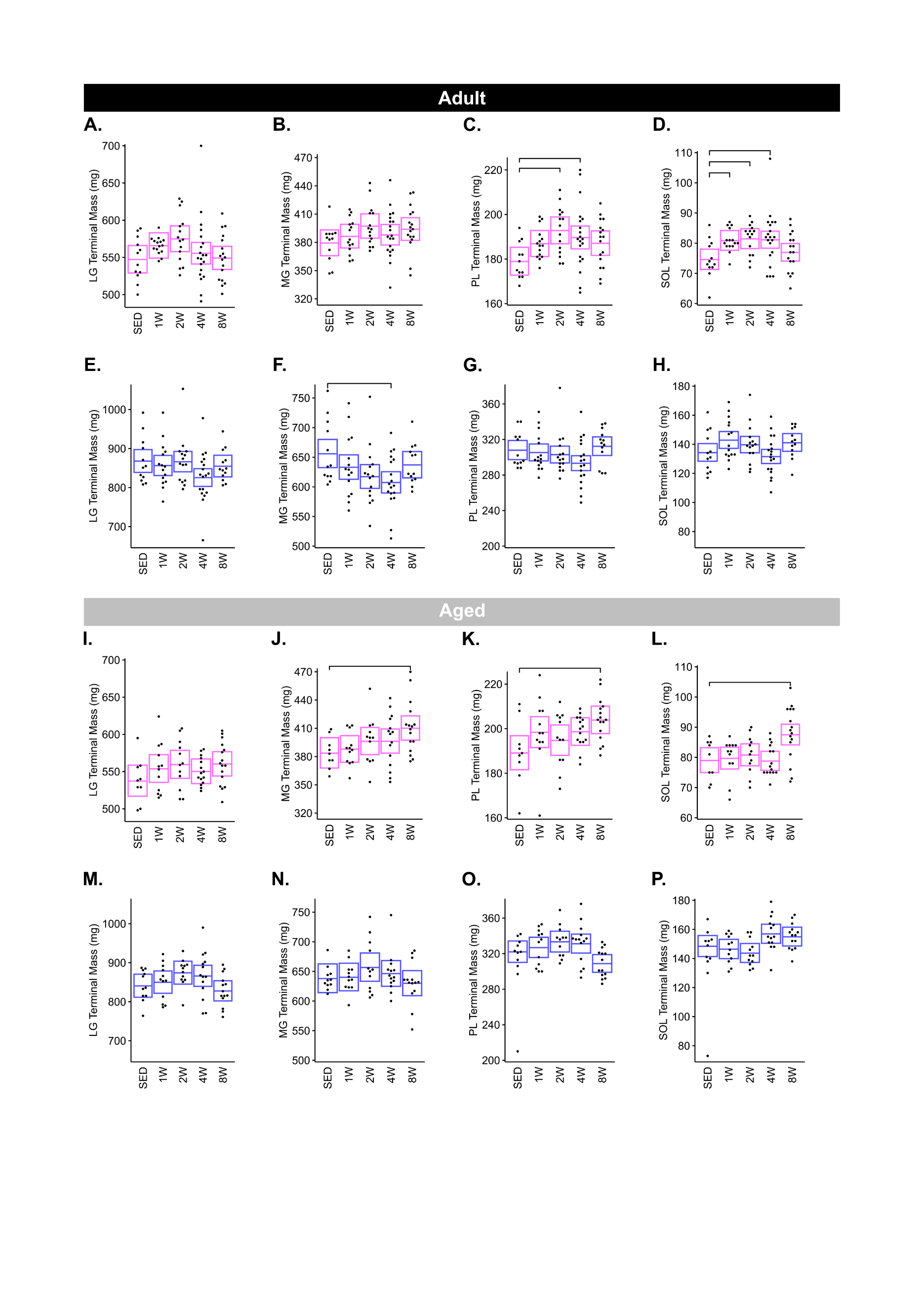
**

**Figure S7. Terminal muscle mass.** Terminal masses of LG, MG, PL, and SOL muscles in Adult females (**A–C**), Adult males (**D–F**), Aged females (**G–I**), and Aged males (**J–L**). Boxes are 95% confidence intervals for the mean mass of each group, and they are colored by sex (female=pink, male=blue). Two-sided *t*-tests were used to compare each trained group to SED, and brackets indicate a statistically significant difference between groups (Holm p < 0.05).

**
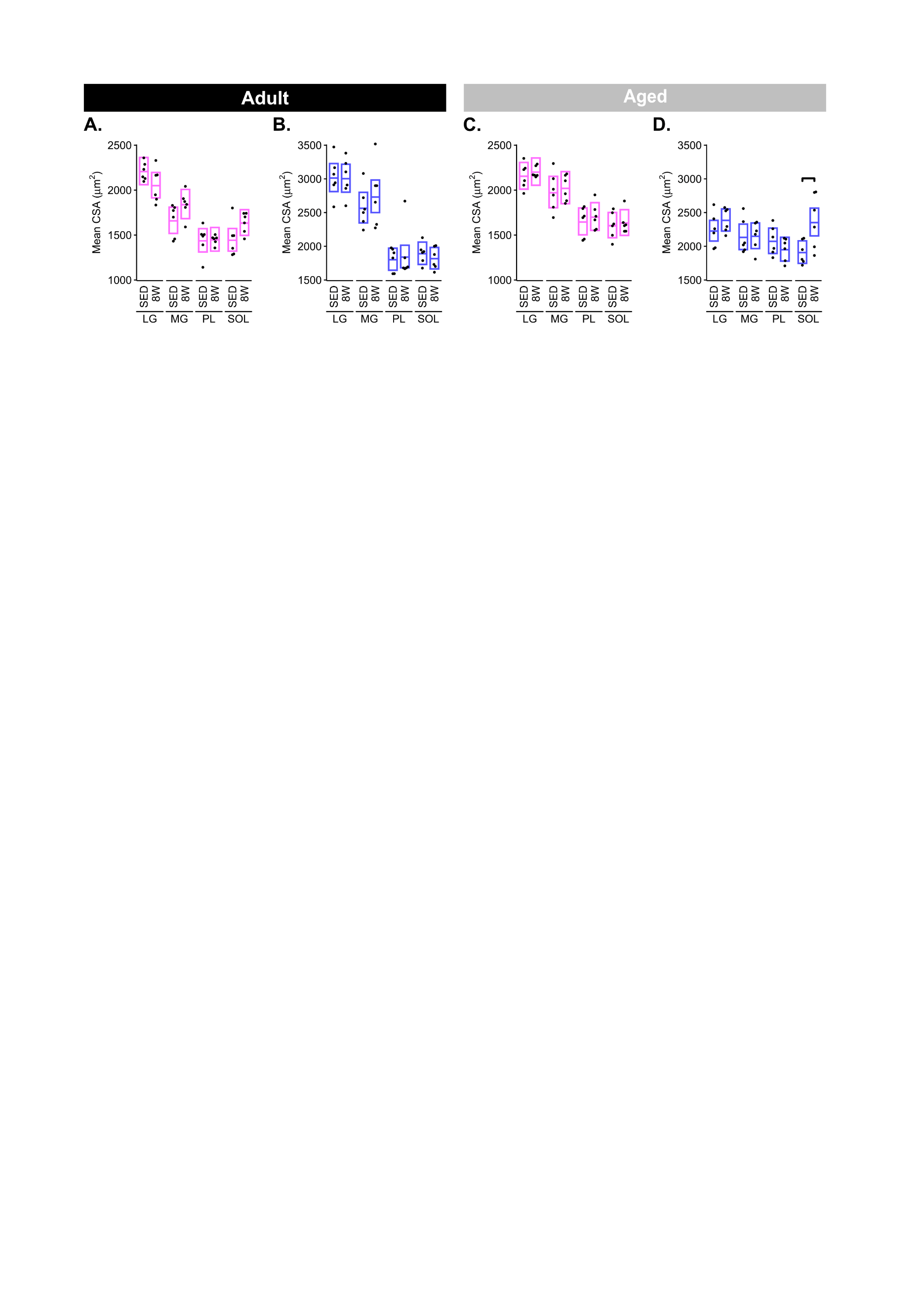
**

**Figure S8. Mean muscle fiber CSA.** Mean cross-sectional area (CSA) across fiber types for the LG, MG, PL, and SOL muscles from Adult females (**A**), Adult males (**B**), Aged females (**C**), and Aged males (**D**). Boxes are 95% confidence intervals for the mean CSA of each group, and they are colored by sex (female=pink, male=blue). Student’s *t*-tests were used to compare 8W to SED, and brackets indicate a statistically significant difference between groups (Holm p < 0.05).

**
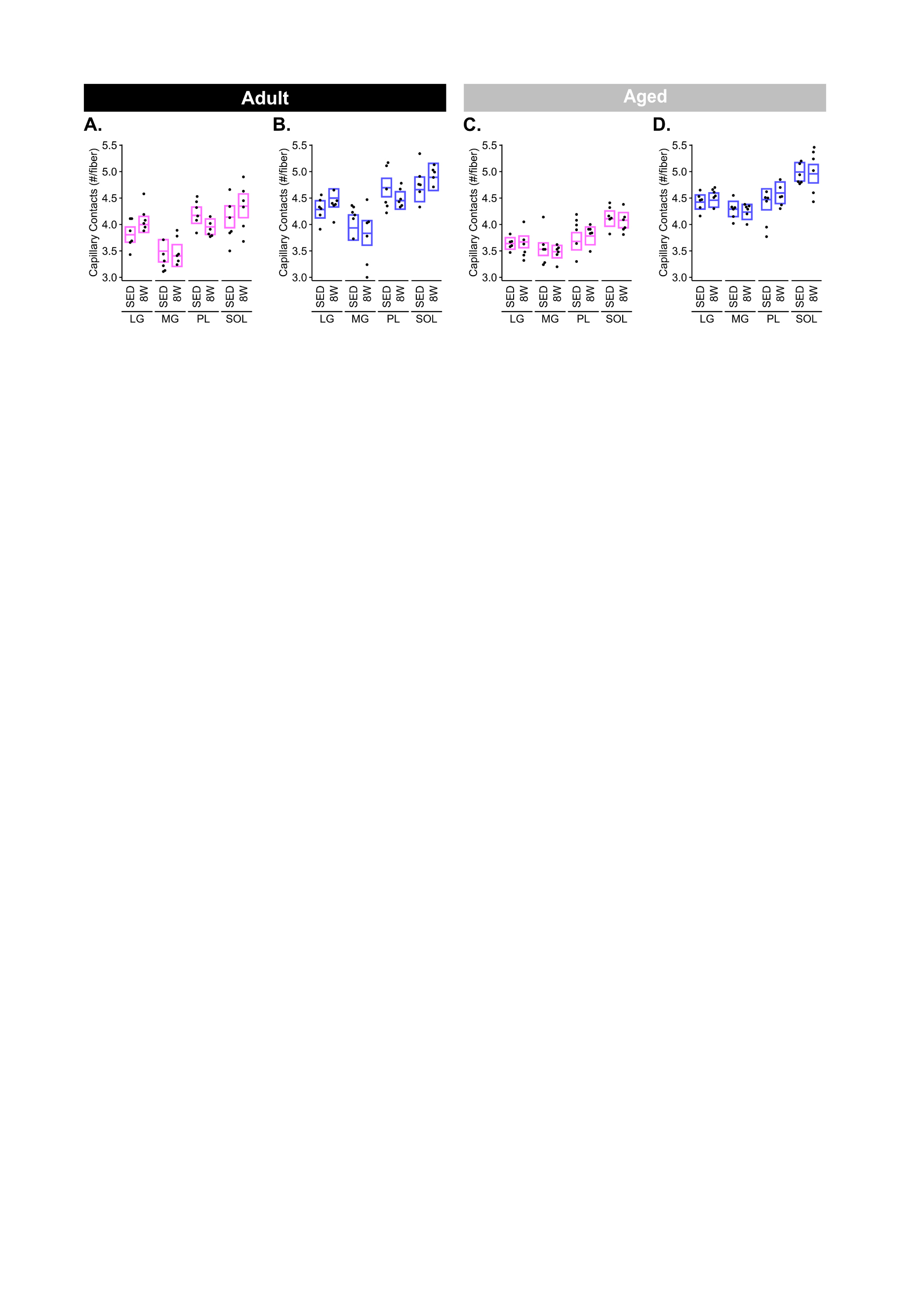
**

**Figure S9. Capillary contacts (mean).** Mean number of capillary contacts per fiber for the LG, MG, PL, and SOL muscles from Adult females (**A**), Adult males (**B**), Aged females (**C**), and Aged males (**D**). Boxes are 95% confidence intervals for the mean of each group, and they are colored by sex (female=pink, male=blue). For each muscle, the 8W trained group was compared to SED. None of the comparisons were significant at the 0.05 level.
