# Supplementary material for: Physiological Adaptations to Progressive Endurance Exercise Training in Adult and Aged Rats: Insights from the Molecular Transducers of Physical Activity Consortium (MoTrPAC)
Source: Function (Oxf). 2024 Mar 28;5(4):zqae014. doi: 10.1093/function/zqae014 (PMC11245678; doi:10.1093/function/zqae014)
Supplement: zqae014_Supplemental_Files [file zqae014_supplemental_files.zip › Table S1.docx]

**Table S1. Animal allocation and usage.** Sedentary (SED), Training Weeks (W), Male (M), Female (F).

| **A** | **B** | **C** | **D** | **E** | **F** | **G** | **H** |
| --- | --- | --- | --- | --- | --- | --- | --- |
| **Arrival Date (mon/year)** | **Arrival Age**  **(months)** | **Sex** | **# Rats**  **Arrival** | **# Rats**  **Compliant** | **# Rats**  **Completed** | **Groups** | **Testing Date**  **(month/year)** |
| 6/2018 | 5 | M | 30 | 28 | 12  13* | SED  8W | 7-8/2018 |
| 7/2018 | 5 | F | 30 | 30 | 12  17 ** | SED  8W | 8-9/2018 |
| 8/2018 | 4 | M | 20 | 18 | 18 | 4W | 10/2018 |
| 9/2018 | 4 | F | 20 | 20 | 20 | 4W | 11/2018 |
| 10/2018 | 3 | M | 30 | 30 | 15  15 | 1W  2W | 1/2019 |
| 11/2018 | 3 | F | 30 | 30 | 15  15 | 1W  2W | 2/2019 |
|  |  |  |  |  |  |  |  |
| 1/2019 | 17 | M | 30 | 28 | 9  15 | SED  8W | 2-3/2019 |
| 2/2019 | 17 | F | 30 | 29 | 10##  16 | SED  8W | 3-4/2019 |
| 3/2019 | 16 | M | 20 | 18 | 14^  3 | 4W  SED (8W) | 5/2019 |
| 4/2019 | 16 | F | 20 | 20 | 16^^ | 4W | 6/2019 |
| 5/2019 | 15 | M | 30 | 30 | 5&  12  12 | SED (1W)  1W  2W | 8/2019 |
| 6/2019 | 15 | F | 30 | 30 | 6  12  12 | SED (1W)  1W  2W | 9/2019 |

Animals arrived in twelve cohorts of 20-30 rats over a period of one year (06/2018 to 06/2019). Male (M) and female (F) rats arrived in separate cohorts and were tested separately (see testing dates). The number of rats that arrived, were found to be compliant following familiarization and completed the training is provided in columns D and E, and F, respectively of the table. The number of rats that completed the experiment in their assigned group (sedentary (SED) and trained for 1-week (1W), 2-weeks (2W), 4-weeks (4W) or 8-weeks (8-W)) is provided in columns F and G.

The reason for animal dropouts was:

*****, 3 males did not complete the 8W training (2 had foot injuries while training and 1 died of unknown cause),

******, 1 female did not complete the 8W training due to a foot injury,

**#**; loss of 4 SED males: 1 male stopped running, 1 male with tumors, 2 males died of unknown cause,

**##**, loss of 3 SED females: 1 female died of unknown cause, 2 females with tumors

**^**, 1 male did not complete 4W of training due to tumors

**^^**, 4 females did not complete 4W of training due to tumors

**&**, loss of 1 SED male that died of unknown cause
