# Supplementary material for: Physiological Adaptations to Progressive Endurance Exercise Training in Adult and Aged Rats: Insights from the Molecular Transducers of Physical Activity Consortium (MoTrPAC)
Source: Function (Oxf). 2024 Mar 28;5(4):zqae014. doi: 10.1093/function/zqae014 (PMC11245678; doi:10.1093/function/zqae014)
Supplement: zqae014_Supplemental_Files [file zqae014_supplemental_files.zip › Table S2.docx]

**Table S2. Clinical analyte assay details.**

| **Analyte** | **Catalog No.** | **Company** |
| --- | --- | --- |
| Corticosterone | 55-CORMS-E01 | Alpco (Salem, NH) |
| Glucose | B24985 | Beckman Coulter (Brea, CA) |
| Glycerol | 445850 |  |
| Lactate | A95550 |  |
| Total ketones | 415-73301, 411-73401 | Fujifilm Wako (Osaka, Japan) |
| NEFA | 995-34791, 999-34691, 993-35191, 991-34891 |  |
| Glucagon | K1535YK | Meso Scale Discovery (Rockville, MD) |
| Insulin, Leptin | K15158C (multiplex assay) |  |
