# Supplementary material for: Physiological Adaptations to Progressive Endurance Exercise Training in Adult and Aged Rats: Insights from the Molecular Transducers of Physical Activity Consortium (MoTrPAC)
Source: Function (Oxf). 2024 Mar 28;5(4):zqae014. doi: 10.1093/function/zqae014 (PMC11245678; doi:10.1093/function/zqae014)
Supplement: zqae014_Supplemental_Files [file zqae014_supplemental_files.zip › Table S5 - VO2max (absolute).docx]

**Table S5. Descriptive statistics for absolute (L/min) maximum oxygen consumption (VO_2_max).**

| **Group** | **Timepoint** | **N** | **Mean** | **SD** | **CV** | **Min** | **Max** | **Range** |
| --- | --- | --- | --- | --- | --- | --- | --- | --- |
| Female, Adult, SED | PRE | 12 | 13.15 | 1.15 | 8.7 | 10.98 | 14.94 | 3.96 |
|  | POST | 12 | 13.28 | 1.17 | 8.8 | 11.78 | 15.17 | 3.39 |
| Female, Adult, 4W | PRE | 20 | 13.82 | 0.76 | 5.5 | 12.59 | 15.63 | 3.04 |
|  | POST | 20 | 14.32 | 1.05 | 7.3 | 12.83 | 16.61 | 3.78 |
| Female, Adult, 8W | PRE | 17 | 13.05 | 1.21 | 9.3 | 11.12 | 15.18 | 4.06 |
|  | POST | 17 | 15.64 | 0.79 | 5.1 | 13.75 | 17.17 | 3.42 |
|  | | | | | | | | |
| Male, Adult, SED | PRE | 12 | 21.17 | 1.43 | 6.8 | 19.49 | 24.42 | 4.93 |
|  | POST | 12 | 21.10 | 0.89 | 4.2 | 19.44 | 22.83 | 3.39 |
| Male, Adult, 4W | PRE | 18 | 20.82 | 1.73 | 8.3 | 18.00 | 24.01 | 6.01 |
|  | POST | 18 | 21.62 | 1.73 | 8.0 | 19.33 | 24.69 | 5.36 |
| Male, Adult, 8W | PRE | 13 | 22.09 | 0.93 | 4.2 | 20.11 | 23.37 | 3.20 |
|  | POST | 13 | 24.59 | 1.29 | 5.3 | 22.57 | 26.41 | 3.84 |
|  | | | | | | | | |
| Female, Aged, SED | PRE | 10 | 12.36 | 1.00 | 8.1 | 11.06 | 14.35 | 3.29 |
|  | POST | 10 | 12.31 | 1.45 | 11.8 | 10.90 | 15.96 | 5.06 |
| Female, Aged, 8W | PRE | 16 | 12.89 | 0.73 | 5.6 | 11.12 | 14.20 | 3.08 |
|  | POST | 16 | 14.83 | 0.76 | 5.2 | 13.73 | 16.00 | 2.27 |
|  | | | | | | | | |
| Male, Aged, SED | PRE | 8 | 20.32 | 1.35 | 6.6 | 18.62 | 22.76 | 4.14 |
|  | POST | 8 | 16.60 | 1.06 | 5.4 | 17.77 | 20.71 | 2.94 |
| Male, Aged, 8W | PRE | 15 | 20.00 | 1.06 | 5.3 | 17.77 | 21.90 | 4.13 |
|  | POST | 15 | 21.16 | 1.26 | 5.9 | 17.86 | 23.26 | 5.40 |
