# Supplementary material for: Physiological Adaptations to Progressive Endurance Exercise Training in Adult and Aged Rats: Insights from the Molecular Transducers of Physical Activity Consortium (MoTrPAC)
Source: Function (Oxf). 2024 Mar 28;5(4):zqae014. doi: 10.1093/function/zqae014 (PMC11245678; doi:10.1093/function/zqae014)
Supplement: zqae014_Supplemental_Files [file zqae014_supplemental_files.zip › Table S6 - VO2max (relative).docx]

**Table S6. Descriptive statistics for relative (mL/kg/min) maximum oxygen consumption (VO_2_max).**

| **Group** | **Timepoint** | **N** | **Mean** | **SD** | **CV** | **Min** | **Max** | **Range** |
| --- | --- | --- | --- | --- | --- | --- | --- | --- |
| Female, Adult, SED | PRE | 12 | 73.8 | 5.1 | 6.9 | 65.8 | 83.8 | 18.0 |
|  | POST | 12 | 66.9 | 5.1 | 7.3 | 61.4 | 73.6 | 12.2 |
| Female, Adult, 4W | PRE | 20 | 74.1 | 3.3 | 4.4 | 68.0 | 79.0 | 11.0 |
|  | POST | 20 | 75.9 | 4.2 | 5.5 | 71.7 | 88.4 | 16.7 |
| Female, Adult, 8W | PRE | 17 | 72.6 | 4.8 | 6.7 | 64.4 | 79.6 | 15.2 |
|  | POST | 17 | 82.9 | 2.7 | 3.3 | 76.2 | 88.2 | 12.0 |
|  | | | | | | | | |
| Male, Adult, SED | PRE | 12 | 62.9 | 4.2 | 6.7 | 54.7 | 67.8 | 13.0 |
|  | POST | 12 | 59.2 | 3.5 | 5.9 | 52.6 | 63.5 | 10.9 |
| Male, Adult, 4W | PRE | 18 | 65.4 | 3.0 | 4.6 | 59.9 | 71.0 | 11.1 |
|  | POST | 18 | 68.1 | 2.5 | 3.7 | 64.1 | 72.3 | 8.2 |
| Male, Adult, 8W | PRE | 13 | 66.0 | 3.1 | 4.7 | 60.4 | 72.6 | 12.2 |
|  | POST | 13 | 77.2 | 3.9 | 5.1 | 69.3 | 81.5 | 12.2 |
|  | | | | | | | | |
| Female, Aged, SED | PRE | 10 | 51.6 | 4.9 | 9.5 | 46.2 | 64.3 | 18.1 |
|  | POST | 10 | 51.3 | 7.9 | 15.5 | 44.7 | 72.2 | 27.5 |
| Female, Aged, 8W | PRE | 16 | 53.8 | 3.1 | 5.8 | 49.3 | 59.4 | 10.1 |
|  | POST | 16 | 63.6 | 2.9 | 4.6 | 57.9 | 67.8 | 9.9 |
|  | | | | | | | | |
| Male, Aged, SED | PRE | 8 | 46.5 | 2.9 | 6.3 | 43.4 | 52.0 | 8.6 |
|  | POST | 8 | 46.7 | 2.1 | 4.5 | 46.7 | 49.6 | 6.3 |
| Male, Aged, 8W | PRE | 15 | 46.1 | 2.0 | 4.4 | 41.3 | 50.1 | 8.8 |
|  | POST | 15 | 54.5 | 3.6 | 6.6 | 47.9 | 61.9 | 14.0 |
