# Supplementary material for: Physiological Adaptations to Progressive Endurance Exercise Training in Adult and Aged Rats: Insights from the Molecular Transducers of Physical Activity Consortium (MoTrPAC)
Source: Function (Oxf). 2024 Mar 28;5(4):zqae014. doi: 10.1093/function/zqae014 (PMC11245678; doi:10.1093/function/zqae014)
Supplement: zqae014_Supplemental_Files [file zqae014_supplemental_files.zip › Table S7 - Body Mass.docx]

**Table S7. Descriptive statistics for body mass (grams), which was measured on the day of NMR analysis.**

| **Group** | **Timepoint** | **N** | **Mean** | **SD** | **CV** | **Min** | **Max** | **Range** |
| --- | --- | --- | --- | --- | --- | --- | --- | --- |
| Female, Adult, SED | PRE | 12 | 179.3 | 6.9 | 3.8 | 168.4 | 189.1 | 20.7 |
|  | POST | 12 | 198.6 | 10.1 | 5.1 | 183.0 | 216.0 | 33.0 |
| Female, Adult, 4wk | PRE | 20 | 181.7 | 12.3 | 6.8 | 158.4 | 164.0 | 53.9 |
|  | POST | 20 | 188.8 | 12.6 | 6.6 | 164.0 | 229.1 | 65.1 |
| Female, Adult, 8wk | PRE | 17 | 180.1 | 8.8 | 4.9 | 167.1 | 196.0 | 28.9 |
|  | POST | 17 | 188.8 | 8.6 | 4.5 | 176.0 | 204.0 | 28.0 |
|  | | | | | | | | |
| Male, Adult, SED | PRE | 12 | 335.8 | 19.2 | 5.7 | 304.0 | 371.4 | 67.4 |
|  | POST | 12 | 357.5 | 21.2 | 5.9 | 329.0 | 396.0 | 67.0 |
| Male, Adult, 4wk | PRE | 18 | 322.6 | 25.4 | 7.9 | 272.0 | 354.0 | 82.0 |
|  | POST | 18 | 317.7 | 24.1 | 7.6 | 272.9 | 357.6 | 84.7 |
| Male, Adult, 8wk | PRE | 13 | 334.4 | 13.8 | 4.1 | 304.7 | 335.0 | 46.0 |
|  | POST | 13 | 318.7 | 14.9 | 4.7 | 289.0 | 335.0 | 46.0 |
|  | | | | | | | | |
| Female, Aged, SED | PRE | 10 | 243.2 | 12.3 | 5.1 | 226.8 | 264.3 | 37.5 |
|  | POST | 10 | 241.2 | 12.5 | 5.2 | 221.0 | 264.9 | 43.9 |
| Female, Aged, 4wk | PRE | 16 | 229.2 | 15.3 | 6.7 | 206.9 | 272.0 | 65.1 |
|  | POST | 16 | 224.9 | 14.6 | 6.5 | 205.6 | 264.0 | 58.4 |
| Female, Aged, 8wk | PRE | 16 | 240.0 | 11.1 | 4.6 | 216.6 | 263.8 | 47.2 |
|  | POST | 16 | 233.5 | 9.3 | 4.0 | 217.3 | 261.6 | 44.3 |
|  | | | | | | | | |
| Male, Aged, SED | PRE | 11 | 440.1 | 17.3 | 3.9 | 412.4 | 467.5 | 55.1 |
|  | POST | 11 | 419.4 | 14.7 | 3.5 | 394.2 | 443.9 | 49.7 |
| Male, Aged, 4wk | PRE | 14 | 431.0 | 13.5 | 3.6 | 407.9 | 451.1 | 43.3 |
|  | POST | 14 | 404.6 | 16.4 | 4.1 | 383.0 | 430.3 | 47.3 |
| Male, Aged, 8wk | PRE | 15 | 435.7 | 15.2 | 3.5 | 409.7 | 454.4 | 44.7 |
|  | POST | 15 | 386.9 | 14.5 | 3.7 | 360.0 | 416.4 | 56.4 |
