# Supplementary material for: Physiological Adaptations to Progressive Endurance Exercise Training in Adult and Aged Rats: Insights from the Molecular Transducers of Physical Activity Consortium (MoTrPAC)
Source: Function (Oxf). 2024 Mar 28;5(4):zqae014. doi: 10.1093/function/zqae014 (PMC11245678; doi:10.1093/function/zqae014)
Supplement: zqae014_Supplemental_Files [file zqae014_supplemental_files.zip › Table S8 - Lean Mass.docx]

**Table S8. Descriptive statistics for NMR-derived lean mass (grams).**

| **Group** | **Timepoint** | **N** | **Mean** | **SD** | **CV** | **Min** | **Max** | **Range** |
| --- | --- | --- | --- | --- | --- | --- | --- | --- |
| Female, Adult, SED | PRE | 12 | 105.5 | 4.3 | 4.1 | 98.8 | 113.8 | 15.0 |
|  | POST | 12 | 111.2 | 5.2 | 4.7 | 102.5 | 120.1 | 17.6 |
| Female, Adult, 4wk | PRE | 20 | 106.0 | 6.8 | 6.4 | 92.5 | 124.2 | 31.7 |
|  | POST | 20 | 112.6 | 7.1 | 6.3 | 97.1 | 132.0 | 34.9 |
| Female, Adult, 8wk | PRE | 17 | 105.2 | 4.9 | 4.7 | 96.0 | 113.6 | 17.6 |
|  | POST | 17 | 112.5 | 4.5 | 4.0 | 105.9 | 120.2 | 14.3 |
|  | | | | | | | | |
| Male, Adult, SED | PRE | 12 | 189.1 | 10.4 | 5.5 | 173.6 | 208.0 | 34.4 |
|  | POST | 12 | 200.8 | 12.9 | 6.4 | 182.8 | 222.8 | 40.0 |
| Male, Adult, 4wk | PRE | 18 | 177.3 | 14.3 | 8.0 | 150.4 | 196.8 | 46.4 |
|  | POST | 18 | 182.6 | 14.3 | 7.8 | 158.1 | 204.9 | 46.8 |
| Male, Adult, 8wk | PRE | 13 | 188.6 | 7.5 | 4.0 | 169.4 | 195.6 | 26.2 |
|  | POST | 13 | 188.2 | 10.3 | 5.5 | 164.3 | 199.7 | 35.4 |
|  | | | | | | | | |
| Female, Aged, SED | PRE | 10 | 135.0 | 5.8 | 4.3 | 129.5 | 145.1 | 15.6 |
|  | POST | 10 | 127.5 | 5.4 | 4.2 | 121.4 | 138.0 | 16.6 |
| Female, Aged, 4wk | PRE | 16 | 123.2 | 6.4 | 5.2 | 113.2 | 140.6 | 27.4 |
|  | POST | 16 | 119.6 | 5.9 | 4.9 | 110.0 | 134.9 | 24.9 |
| Female, Aged, 8wk | PRE | 16 | 135.6 | 5.8 | 4.3 | 127.5 | 149.6 | 22.1 |
|  | POST | 16 | 127.8 | 4.5 | 3.6 | 120.4 | 136.8 | 16.4 |
|  | | | | | | | | |
| Male, Aged, SED | PRE | 11 | 229.5 | 7.2 | 3.2 | 217.3 | 239.4 | 22.1 |
|  | POST | 11 | 219.8 | 7.3 | 3.3 | 208.1 | 229.8 | 21.7 |
| Male, Aged, 4wk | PRE | 14 | 226.1 | 6.5 | 2.9 | 210.5 | 235.8 | 25.3 |
|  | POST | 14 | 219.1 | 6.4 | 2.9 | 206.9 | 230.2 | 23.3 |
| Male, Aged, 8wk | PRE | 15 | 226.9 | 7.2 | 3.2 | 213.9 | 236.8 | 22.9 |
|  | POST | 15 | 212.0 | 6.7 | 3.1 | 200.2 | 222.8 | 22.6 |
