# Supplementary material for: Physiological Adaptations to Progressive Endurance Exercise Training in Adult and Aged Rats: Insights from the Molecular Transducers of Physical Activity Consortium (MoTrPAC)
Source: Function (Oxf). 2024 Mar 28;5(4):zqae014. doi: 10.1093/function/zqae014 (PMC11245678; doi:10.1093/function/zqae014)
Supplement: zqae014_Supplemental_Files [file zqae014_supplemental_files.zip › Table S9 - Fat Mass.docx]

**Table S9. Descriptive statistics for NMR-derived fat mass (grams).**

| **Group** | **Timepoint** | **N** | **Mean** | **SD** | **CV** | **Min** | **Max** | **Range** |
| --- | --- | --- | --- | --- | --- | --- | --- | --- |
| Female, Adult, SED | PRE | 12 | 20.4 | 2.1 | 10.0 | 16.0 | 24.0 | 8.0 |
|  | POST | 12 | 28.6 | 3.5 | 12.1 | 22.9 | 34.3 | 11.4 |
| Female, Adult, 4wk | PRE | 20 | 21.3 | 3.7 | 17.5 | 10.9 | 28.2 | 17.3 |
|  | POST | 20 | 21.34 | 3.6 | 17.0 | 12.4 | 31.8 | 19.4 |
| Female, Adult, 8wk | PRE | 17 | 19.7 | 2.9 | 14.7 | 15.2 | 24.5 | 9.3 |
|  | POST | 17 | 20.6 | 3.7 | 17.8 | 16.3 | 29.7 | 13.4 |
|  | | | | | | | | |
| Male, Adult, SED | PRE | 12 | 47.5 | 4.3 | 9.1 | 39.8 | 53.9 | 14.1 |
|  | POST | 12 | 55.2 | 8.8 | 15.9 | 44.7 | 75.5 | 30.8 |
| Male, Adult, 4wk | PRE | 18 | 45.4 | 5.9 | 13.1 | 36.4 | 58.5 | 22.1 |
|  | POST | 18 | 37.4 | 5.0 | 13.3 | 29.6 | 49.7 | 20.1 |
| Male, Adult, 8wk | PRE | 13 | 46.6 | 5.5 | 11.7 | 38.8 | 57.5 | 18.7 |
|  | POST | 13 | 29.1 | 5.4 | 18.7 | 19.3 | 40.8 | 21.5 |
|  | | | | | | | | |
| Female, Aged, SED | PRE | 10 | 47.6 | 5.9 | 12.4 | 37.9 | 56.9 | 19.0 |
|  | POST | 10 | 44.9 | 6.9 | 15.3 | 34.1 | 53.7 | 19.6 |
| Female, Aged, 4wk | PRE | 16 | 40.8 | 7.0 | 17.2 | 32.7 | 61.7 | 29.0 |
|  | POST | 16 | 36.4 | 6.8 | 18.6 | 28.4 | 56.0 | 27.6 |
| Female, Aged, 8wk | PRE | 16 | 43.7 | 4.7 | 10.9 | 31.0 | 50.7 | 19.7 |
|  | POST | 16 | 37.5 | 2.9 | 7.8 | 33.5 | 45.5 | 12.0 |
|  | | | | | | | | |
| Male, Aged, SED | PRE | 11 | 82.5 | 10.2 | 12.4 | 67.1 | 100.0 | 32.9 |
|  | POST | 11 | 72.4 | 8.3 | 11.4 | 62.4 | 87.2 | 24.8 |
| Male, Aged, 4wk | PRE | 14 | 77.0 | 6.9 | 9.0 | 64.3 | 88.6 | 24.3 |
|  | POST | 14 | 61.5 | 7.3 | 12.0 | 50.2 | 73.2 | 23.0 |
| Male, Aged, 8wk | PRE | 15 | 81.3 | 7.7 | 9.5 | 70.5 | 95.0 | 24.5 |
|  | POST | 15 | 53.9 | 7.4 | 13.7 | 43.6 | 70.8 | 27.2 |
